# Protective role of the vulture facial skin and gut microbiomes aid adaptation to scavenging

**DOI:** 10.1186/s13028-018-0415-3

**Published:** 2018-10-11

**Authors:** Marie Lisandra Zepeda Mendoza, Michael Roggenbuck, Karla Manzano Vargas, Lars Hestbjerg Hansen, Søren Brunak, M. Thomas P. Gilbert, Thomas Sicheritz-Pontén

**Affiliations:** 1 Centre for GeoGenetics, Natural History Museum of Denmark, University of Copenhagen, Øster Voldgade 5-7, 1350 Copenhagen K, Denmark; 2 Department for Bioinformatics and Microbe Technology, Novozymes A/S, 2880 Bagsværd, Denmark; 3 Undergraduate Program on Genomic Sciences, Center for Genomic Sciences, National Autonomous University of Mexico, Av. Universidad s/n Col. Chamilpa, 62210 Cuernavaca, Morelos Mexico; 4 Section for Microbiology and Biotechnology, Department of Environmental Science, Aarhus University, Frederiksborgvej 399, 4000 Roskilde, Denmark; 5 Center for Biological Sequence Analysis, Department of Bio and Health Informatics, Technical University of Denmark, Anker Engelunds Vej 1 Bygning 101A, 2800 Kgs. Lyngby, Denmark; 6 Novo Nordisk Foundation Center for Protein Research, Faculty of Health and Medical Sciences, University of Copenhagen, Blegdamsvej 3, 2200 Copenhagen N, Denmark; 7 Norwegian University of Science and Technology, University Museum, 7491 Trondheim, Norway; 8 Centre of Excellence for Omics-Driven Computational Biodiscovery (COMBio), Faculty of Applied Sciences, AIMST University, 08100 Bedong, Malaysia

**Keywords:** Colonization resistance, Diet specialization, Metagenomics, Microbiome, Pathogens, Scavenging, Vulture

## Abstract

**Background:**

Vultures have adapted the remarkable ability to feed on carcasses that may contain microorganisms that would be pathogenic to most other animals. The holobiont concept suggests that the genetic basis of such adaptation may not only lie within their genomes, but additionally in their associated microbes. To explore this, we generated shotgun DNA sequencing datasets of the facial skin and large intestine microbiomes of the black vulture (*Coragyps atratus*) and the turkey vulture (*Cathartes aura*). We characterized the functional potential and taxonomic diversity of their microbiomes, the potential pathogenic challenges confronted by vultures, and the microbial taxa and genes that could play a protective role on the facial skin and in the gut.

**Results:**

We found microbial taxa and genes involved in diseases, such as dermatitis and pneumonia (more abundant on the facial skin), and gas gangrene and food poisoning (more abundant in the gut). Interestingly, we found taxa and functions with potential for playing beneficial roles, such as antilisterial bacteria in the gut, and genes for the production of antiparasitics and insecticides on the facial skin. Based on the identified phages, we suggest that phages aid in the control and possibly elimination, as in phage therapy, of microbes reported as pathogenic to a variety of species. Interestingly, we identified *Adineta vaga* in the gut, an invertebrate that feeds on dead bacteria and protozoans, suggesting a defensive predatory mechanism. Finally, we suggest a colonization resistance role through biofilm formation played by Fusobacteria and Clostridia in the gut.

**Conclusions:**

Our results highlight the importance of complementing genomic analyses with metagenomics in order to obtain a clearer understanding of the host-microbial alliance and show the importance of microbiome-mediated health protection for adaptation to extreme diets, such as scavenging.

**Electronic supplementary material:**

The online version of this article (10.1186/s13028-018-0415-3) contains supplementary material, which is available to authorized users.

## Background

Vultures are composed of two clades of carrion-scavenging birds that diverged more than 60 million years ago [1], the New World vultures (Cathartidae) and Old World vultures (Accipitridae). Vultures global populations are under serious threats due to e.g. collisions with wind-energy turbines [2], their use for traditional medicine [3], or ingestion of lead bullets from deer carcasses [4]. Vultures are known as “nature’s clean-up crew”, as they feed on tissues of animals that have died mainly from malnutrition, accidents, predation, and diseases [5–7]. Vultures are thus exposed to a variety of pathogens, including those that cause anthrax, tuberculosis, and brucellosis. A better understanding of various aspects for their biology are necessary, such as their susceptibility to the pathogens in their diet and their role in the transmission of infectious diseases [8, 9].

Vertebrate carcasses are very nutrient-rich resources. It has been speculated that the release of toxins and pathogenicity genes in the carcass microbiome are part of a microbial strategy for outcompeting other microbes [10, 11]. The main colonizers of a carcass are microbes originating from the microbiome of the animal when alive, some of which might become pathogenic in the carcass environment [12]. Other components of the post-mortem microbiome include soil-dwelling bacteria, nematodes, fungi, and insects [13]. In spite of the potentially serious health implications posed by their consumption, the pathogenic repertoire of the gut of these birds has not been fully characterized in relation to their possible implications in the environment. Thus, one of the most intriguing aspects of vulture biology is how they protect themselves against the health challenges posed by their dietary source. Physiologic, genetic and genomic analyses of different species of vultures have explored this aspect and identified genes associated with respiration, immunity, and gastric secretion as possible adaptations to its scavenging diet [14, 15]. For example, due to the previously reported very low stomach pH of a small sample of Old World vulture species [16], it has been suggested that the vulture stomach acidity serves as a filter of potential pathogens [14, 17].

With the genomic revolution, it has become apparent that besides genomic changes, host-associated microbiota plays an important role in diet specialization across vertebrates [18] and that the gut microbiome may play a highly relevant yet unexplored role in diet-driven speciation [19]. The gut microbiome is intimately related to digestion functions, such as energy harvest, nutrient acquisition, and intestinal homeostasis [20]. It has also been shown that the microbiome plays a health protective role to the host by interacting with the host’s immune system and mediating colonization-resistance against pathogens [21, 22]. Furthermore, disorders in the microbiome can lead to diseases such as irritable bowel syndrome, inflammatory bowel disease, obesity, and diabetes [23–25]. In light of the key roles that host-microbiome relationships can play in adaptation, it has been acknowledged that vulture genomic adaptations alone may not provide the full answer to the vulture adaptation to scavenging [14]. However, neither the complete microbial taxonomic diversity (including non-bacterial microbes) nor the gene catalogue of the microbiome of any vulture species has been examined for their protective role against microbes that would normally pose serious health risks for other non-scavenging vertebrate species.

In order to evaluate the protective role of the vulture’s facial skin and gut microbiome, we generated metagenomic datasets from facial skin swabs and gut samples for two species of New World vultures, the black vulture (*Coragyps atratus*) and the turkey vulture (*Cathartes aura*), and performed taxonomic and functional metagenomic analyses.

## Methods

### Sampling method and DNA sequencing

We generated DNA shotgun metagenome datasets from a subset of the samples used by Roggenbuck et al. [17]. Samples were collected over a period of several days in Tennessee, USA. Black vultures were live-trapped at deer carcasses and then transported to a central facility within a couple of hours of trapping. They were then euthanized with CO_2_, necropsied, and sampled within 30–45 min of death. Turkey vultures were shot at roosts, bagged individually, and transported to the processing facility where they were refrigerated 2–6 h before necropsy and sampling. To collect the gut samples, carcasses were opened to expose the entire gastrointestinal tract. A section of around 3–4 cm of the large intestine (hereafter called gut) located 2–3 cm above the cloaca was isolated with a pair of medical haemostats. Afterwards, 2–3 mL of sterile water was injected through the wall of the intestine with a sterile single-use syringe. The haemostat-blocked section of the intestine was gently massaged with the needle still inserted, and then the wash liquid was aspirated with the syringe. The aspirant was injected into a sterile vial containing RNAlater. Facial skin samples were taken by using sterile polyester swabs saturated with sterile water and wiped across the facial skin of the vultures. Swab tips were cut off and immersed in sterile vials filled with RNAlater. DNA was extracted and the shotgun libraries for HiSeq PE 100 were prepared using the Nextera library building kit following the manufacturer’s instructions, as in Roggenbuck et al. [17]. From the total of 48 different sampled individuals (25 black vultures, and 23 turkey vultures), we used 33 facial skin samples (17 black vultures; 16 turkey vultures) and 47 intestinal samples (25 black vultures; 22 turkey vultures).

### Data processing

Two pipelines were used to process the raw reads. In the first approach, we removed adapter sequences and bases with quality < 15 using Trimmomatic v0.32 [26]. Afterwards, in order to filter out non-bacterial reads derived from the vulture, human, and *Phi* phage (used as control required to increase library complexity on the Illumina HiSeq), the datasets were mapped against the bird genomes dataset of the avian phylogenomic project [27] (which includes the turkey vulture genome), the human (hg19), and the *Phi* phage genomes. Only the non-mapping reads were retained. The second approach was developed to take into account possible *k*-mer bias in the first bases of the reads that could have implications in the subsequent de novo assembly and gene prediction. To this end, we trimmed the first 16 bases of the reads with Trimmomatic v0.32. We then processed those reads with a Metagenomics Assembly and Gene Prediction Toolkit (MOCAT) [28] to clean them of low quality bases and adaptors and screen them versus the turkey vulture, human, and *Phi* phage genomes.

### Taxonomic profiling

We used MGmapper [29] to map with bwa v0.7.10 [30] the filtered cleaned reads against the next databases in full mode: MetaHitAssembly [31], HumanMicrobiome [32], ResFinder [33], Plasmid, Virulence, GreenGenes [34], and Silva [35]. We also mapped in chain mode to the next whole genome databases downloaded from GenBank in the given order: human, plants, vertebrates, invertebrates, protozoa, fungi, and viruses. The remaining non-mapping reads were mapped to the whole genome databases of bacteria. Using the unique mapping reads, we calculated the coverage (percentage of reference sequence covered by reads) of the identified species. The coverage was then used to filter the identifications as follows.

1. Relaxed filtering: In order to ensure the identification of low abundant taxa, we removed identifications with 90% of the abundance signal coming from only three samples.
2. Strict filtering: On top of the relaxed filtering, we removed the species with a coverage lower than the 1st quartile (Qu) value from the coverage distribution of the corresponding database.

With the filtered taxa, we classified the species as present in at least 90% and 50% of all the samples, thus defining a strict and a relaxed, respectively, taxonomic microbiome core.

For each database, we compared the taxa present only in the facial skin or gut datasets, those present in both, and those in significant differential abundance (P value < 0.05). To identify the differentially abundant species, we performed Wilcoxon and t-tests on the normalized abundance distribution of the identified species in the facial skin dataset versus the gut dataset. We also evaluated the taxonomic intra and inter sample variation between the facial skin and gut samples by calculating the Euclidean distances of their normalized abundances using the Ward.D method in R [36].

We then used the identifications of the relaxed filtering to test for microbial abundance correlations by calculating the Spearman correlation for each pairwise comparison of the microbes and calculated the P with a Bonferroni correction on those with a correlation value > 0.8 and < − 0.7. We also examined the enrichment and depletion of taxa within the facial skin and gut microbiome. To this end, we calculated their mean abundance (x̅) across the samples and compared them to the total distribution to calculate the Bonferroni corrected P. From these assignations, we also obtained a specific facial skin and gut core. As before, we defined two types of microbial cores: a strict one that retains those taxonomic identifications at the species level present in at least 80% of the samples of each sample type (facial skin or gut), and a relaxed one that retains those species present in at least 50% of the samples of each sample type.

We also identified the taxa of the top most abundant identified genes, which we defined as those genes with > 2000 mapping reads in the facial skin dataset and > 5000 in the gut dataset. We analysed the principal components (PCs) and rotation matrix of these taxa to identify the ones driving the variation within the facial skin and gut microbiomes. We defined “variation drivers” as those with an absolute rotation matrix value larger than the 3rd Qu value of the distributions from PC1, PC2 and PC3, and as “non-variation drivers” those with less than the 3rd Qu value of the distributions.

Besides the MGmapper identification, we used MOCAT as a complementary taxonomic identification method. For this approach, we used the taxonomic annotation given to the genes from the MOCAT strict non-redundant (NR) gene catalogue. This catalogue contains genes coding for proteins with a minimum length of 80 amino acids, not identified in low abundance, present only bacteria, fungi and virus, and that have an assigned Uniprot annotation (see “Methods”—“Functional profiling”). We analysed these identifications with MEGAN [37] having as input the search of the NR gene set catalogue against Uniprot using Ultra-Fast Sequence Search (USEARCH) [38].

### Pathogenic characterization

We identified potential pathogens in the filtered bacterial and plasmid identifications. To this end, we obtained a list of the bacteria annotated with a disease from the database Pathosystems Resource Integration Center (PATRIC) [39]. In PATRIC, bacteria are annotated as pathogenic if they have been reported with experimental data as causative of a disease. We further added the pathogenicity classification level of bacterial strain using the list from van Belkum [40], which was developed by the Commissie Genetische Modificatie (COGEM). Pathogenicity classes are defined as follows. Class 1 represents species that are commonly non-pathogenic, although there may be differences in virulence among the bacterial strains that should be taken into account. Class 2 contains species that can cause diseases in humans or animals but are unlikely to spread in the human population. Class 3 encompasses species that cause serious human diseases and can disseminate in the human population. We used the metadata of the pathogenic strains obtained from PATRIC to identify whether or not the identified bacteria are capable of sporulation and of antimicrobial resistance, together with the reported disease and host. For the identification of pathogenic plasmids, we used the list from Ho-Sui et al. [41] on the association of virulence factors with genomic islands of pathogenic bacteria.

We then used R v3.1.1 [36] to examine the distribution of the total number of identified pathogenic bacteria. We grouped the samples by (i) vulture species (turkey and black vulture), and (ii) body sampling place (facial skin and gut). We then tested if the x̅ of the distributions were significantly different with a two-tailed and one-tailed (alternate greater) t-test. Next, we examined the number of samples in which each pathogenic bacterial strain, plasmid, resistance gene, and virulence factor was present. In order to get a potentially pathogenic core, we identified those taxa present in 50% (relaxed core) and 90% (strict core) of the samples. We also identified the taxa present only in the facial skin or the gut microbiome.

### Abundance analyses of potential pathogens

In order to analyse the abundance of the potentially pathogenic microbes across the samples, we first rescaled the number of unique mapping reads by their percentage in the sample. We then removed taxa present in low abundance (those with 90% of their signal coming from < 4 samples). To determine if the identified potential pathogenic bacteria of facial skin and gut differed, we used the rescaled counts to build a dendrogram using a hierarchical clustering on the Euclidean distance. Afterwards, we examined which of the retained potentially pathogenic bacteria were present only on the facial skin or in the gut, and which ones were present in both. We then used a t-test to evaluate if the pathogenic bacterial abundance was statistically different by sample type and by vulture species.

### 16S bacterial taxonomic comparison

We compared the taxonomic bacterial identifications from both gut and facial datasets obtained with 16S analyses by Roggenbuck et al. [17] against the bacterial identifications from our metagenomics datasets using MGmapper, the taxonomic annotation of the de novo assembled genes with Uniprot, and the taxonomic identifications from the unmapped reads obtained with Double Index Alignment of Next-Generation Sequencing Data (DIAMOND) [42].

### Functional profiling

We performed de novo assembly with the De Bruijn Graph De Novo Assembler with Uneven Depth sequencing data (IDBA-UD) [43] and predicted genes with Prodigal [44]. Afterwards, we generated a NR gene catalogue with USEARCH [38] by clustering the predicted genes with 90% identity and keeping the centroid sequences. We then searched the NR gene catalogue against Uniprot [45] with Ublast [38]. The resulting identifications were functionally and taxonomically annotated with the use of a customized python script. Finally, we used DIAMOND v0.6.4 [42] blastx to search the unmapped reads against Uniprot, keeping only the best hits for subsequent functional and taxonomic annotation.

For further functional assessment of the genes at the metabolic pathway level, we converted the Uniprot identifiers to the Kyoto Encyclopedia of Genes and Genomes (KEGG) [46] Enzyme Commission (EC) numbers and linked them to their corresponding metabolic pathway. Using the pathway classification information and the gene presence in each sample, we built a matrix for performing principal component analyses (PCA). Using the rotation matrix from the PCA we identified those pathways with an absolute rotation value within the minimum and 1st Qu values of the distributions of the PC1, PC2, and PC3. In order to distinguish the pathways driving most of the variation between the facial skin and gut microbiomes, we identified those pathways for which the absolute rotation value of their PC1, PC2, and PC3 was larger or equal to the 3rd Qu value of their corresponding distributions. We also obtained the Euclidean distances on the rescaled values of the matrices used for the PCAs.

As a second method, we used MOCAT with the Short Oligonucleotide Analysis Package for short-read de novo assembly (SOAPdenovo) v1.05 [47] for assembling the reads cleaned with the approach that removed their first 16 bases. Subsequently, we corrected the assembly for indels and chimeric regions with SOAPdenovo. Using Prodigal, we then predicted the genes from all the samples, pooled them, and built an NR gene catalogue with Uclust [38] using a 90% identity threshold. Facial skin and gut datasets were treated separately. In the MGmapper core definition approach, the NR gene catalogue was obtained for each sample, then the catalogues were pooled and the unique genes were kept to compare their presence or absence across the samples. In contrast, in this approach using MOCAT, we built the cores based on the abundance of the reads mapping to the NR gene catalogue. To this end, we first mapped the reads of each sample against the NR gene catalogue and rescaled the counts values. Then, we removed those genes in low abundance (< 200 mapped reads), without a Uniprot annotation, and not derived from bacteria, archaea, virus, or fungi. We also removed genes coding for proteins with < 80 amino acids aligned to a hit from the Uniprot database. From these proteins, we also obtained a strict core (at least 80% of the samples) and a relaxed core (at least 50% of the samples). We also identified the top most abundant proteins (those with > 2000 mapping reads in the facial skin and > 5000 in the gut samples). On the relaxed functional core, we performed pathway functional analyses of their EC numbers with KEGG.

### Antibiotic resistance

In order to search for antibiotic resistance genes, besides searching the ResFinder database with MGmapper, we used the Resfams v1.2 database [48] and the associated profile hidden Markov models. We searched the de novo assembled NR gene set of each sample against the Resfams profiles with a software for multiple alignment using hidden Markov models (HMMER v3.0) [49].

## Results

### Metagenomic dataset

We produced a total of 342,279,763 raw read pairs from the facial skin samples and 512,803,778 from the gut samples. After cleaning and removing endogenous DNA by mapping against the bird genomes of the avian phylogenomic project [27], we obtained 79,938,910 read pairs from the facial skin samples (with a median of 1,378,000 read pairs per sample) and 144,877,366 from the gut samples (with a median of 1,118,000 per sample) (Additional file 1).

To prove the consistency of the taxonomic profiling between the two vulture species, we compared the number of identified microbial taxa in each species. We filtered the MGmapper [29] identifications of each whole-genome database by depth and breadth (percentage of covered reference sequence) of coverage and identified taxa differentially abundant in the facial skin and gut samples (Additional file 2). The number of identified bacteria was not significantly different between vulture species in the pooled datasets of facial skin and gut samples (P = 0.52, x̅_black vulture_ = 366.97, x̅_turkey vulture_ = 334.65). There were no significant differences between vulture species in the number of identified species of fungi (P = 0.43, x̅_black vulture_ = 9, x̅_turkey vulture_ = 7.5), viruses (P = 0.33, x̅_black vulture_ = 21, x̅_turkey vulture_ = 28.2), plasmids (P = 0.68, x̅_black vulture_ = 186.85, x̅_turkey vulture_ = 173.65), and protozoa (P = 0.21, x̅_black vulture_ = 12, x̅_turkey vulture_ = 9.62). Also, the number of identified proteins with resistance to antibiotics did not differ between vulture species (P = 0.64, x̅_black vulture_ = 107.5, x̅_turkey vulture_ = 100.78).

We compared our metagenomic bacterial identifications to those of Roggenbuck et al. [17]. A total of 735 bacterial operational taxonomic units were identified analysing the 16S sequences, of which 93 were not found among our metagenomics identifications with strict filtering. When using the pre-filtering identifications from the whole-genome bacterial database and those identifications from the GreenGenes [34] and Silva [35] databases, only 14 genera were not identified in our analysis (Additional file 1).

### Taxonomic characterization

Compared to the gut, the facial skin microbiome had higher microbial richness in terms of number of taxa and variation between individuals (P_protozoa_ = 0.021, P_fungi_ = 0.029, P_bacteria_ = 0.0002). However, there was no significant difference in abundance (P_protozoa_ = 0.514, P_fungi_ = 0.47, P_bacteria_ = 0.71). Although the number of identified virus was not significantly different between facial skin and gut (P = 0.58), viruses were statistically less abundant and variable in the facial skin than in the gut samples (P = 0.0002, Euclidean distance_facial skin_ = 9.04, Euclidean distance_gut_ = 13.14). The most abundant bacterial genera in the facial skin microbiome were *Pseudomonas*, *Bacteroides*, and *Prevotella*, while the most abundant in the gut microbiome belonged to *Escherichia*, *Campylobacter*, and *Clostridium* (Additional file 3).

A total of 143 bacterial strains were significantly more abundant within the facial skin microbiome, 46 after the breadth filtering (mostly *Pseudomonas*). Within the gut microbiome, we identified 56 bacterial strains as the highest abundant, 33 after the breadth filtering (mostly *Escherichia* and *Campylobacter*). Bacterial strains most abundant in the facial skin dataset fell into three broad categories: (i) reported as potential human pathogens, (ii) associated with bioremediation (ionizing resistant, reducers of heavy metals, or oil degraders), and (iii) potentially beneficial (producers of antibiotics, insecticides and antifungals), usually intestinal bacteria, and related to water, plants, or soil. Those significantly more abundant in the gut dataset could be classified as: (i) reported as potential human pathogens, (ii) potentially beneficial, mostly intestinal or faecal bacteria from chicken, and (iii) fermenters and producers of intestinal metabolites.

**Fig. 1:**
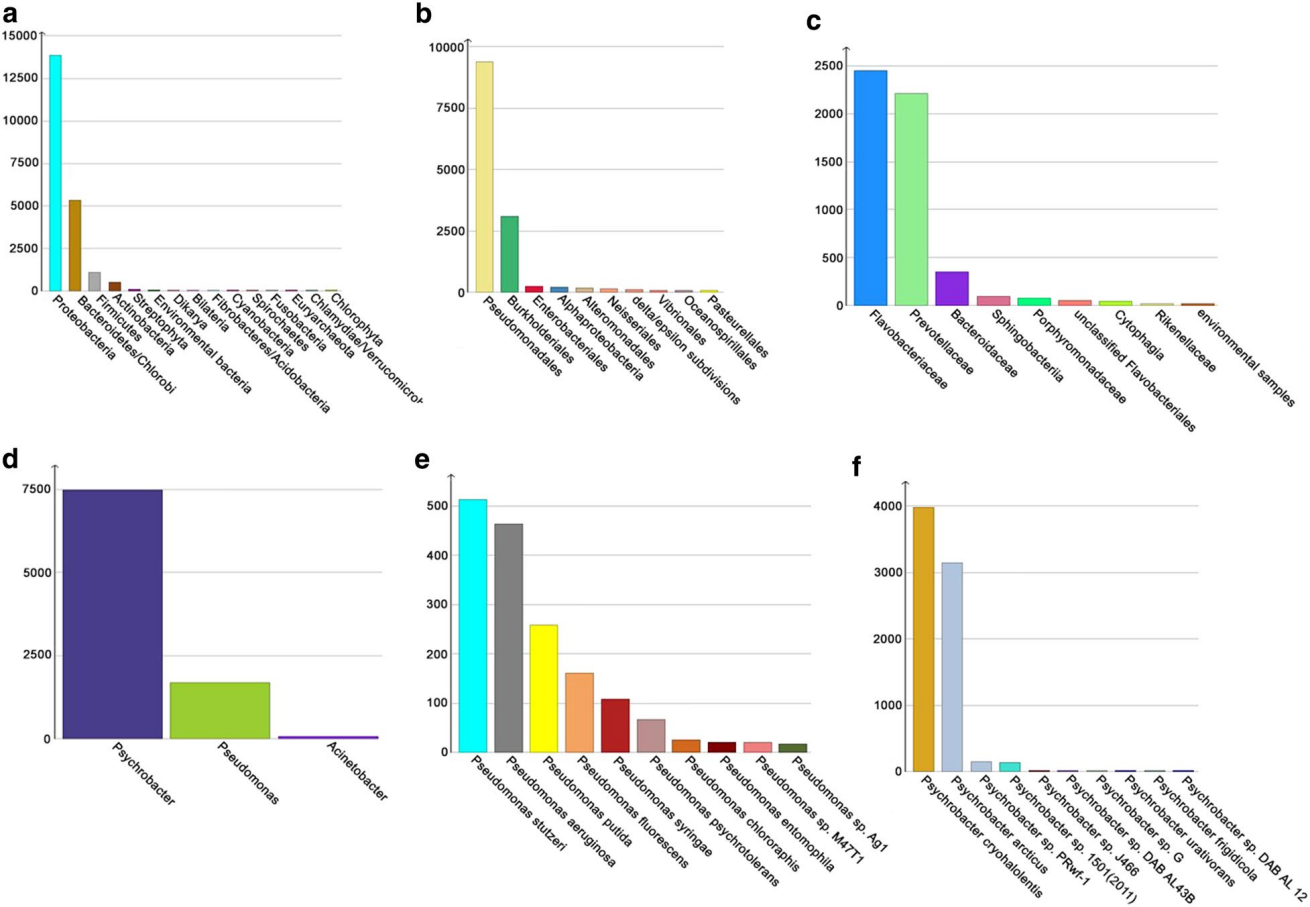
Taxonomic profile of the most abundant facial skin microbiota with MEGAN filtered NR gene catalogue. **a** Phylum level, **b** Proteobacteria, **c** Bacteroidetes, **d** Pseudomonadales, **e** *Pseudomonas*, and **f** *Psychrobacter*

**Fig. 2:**
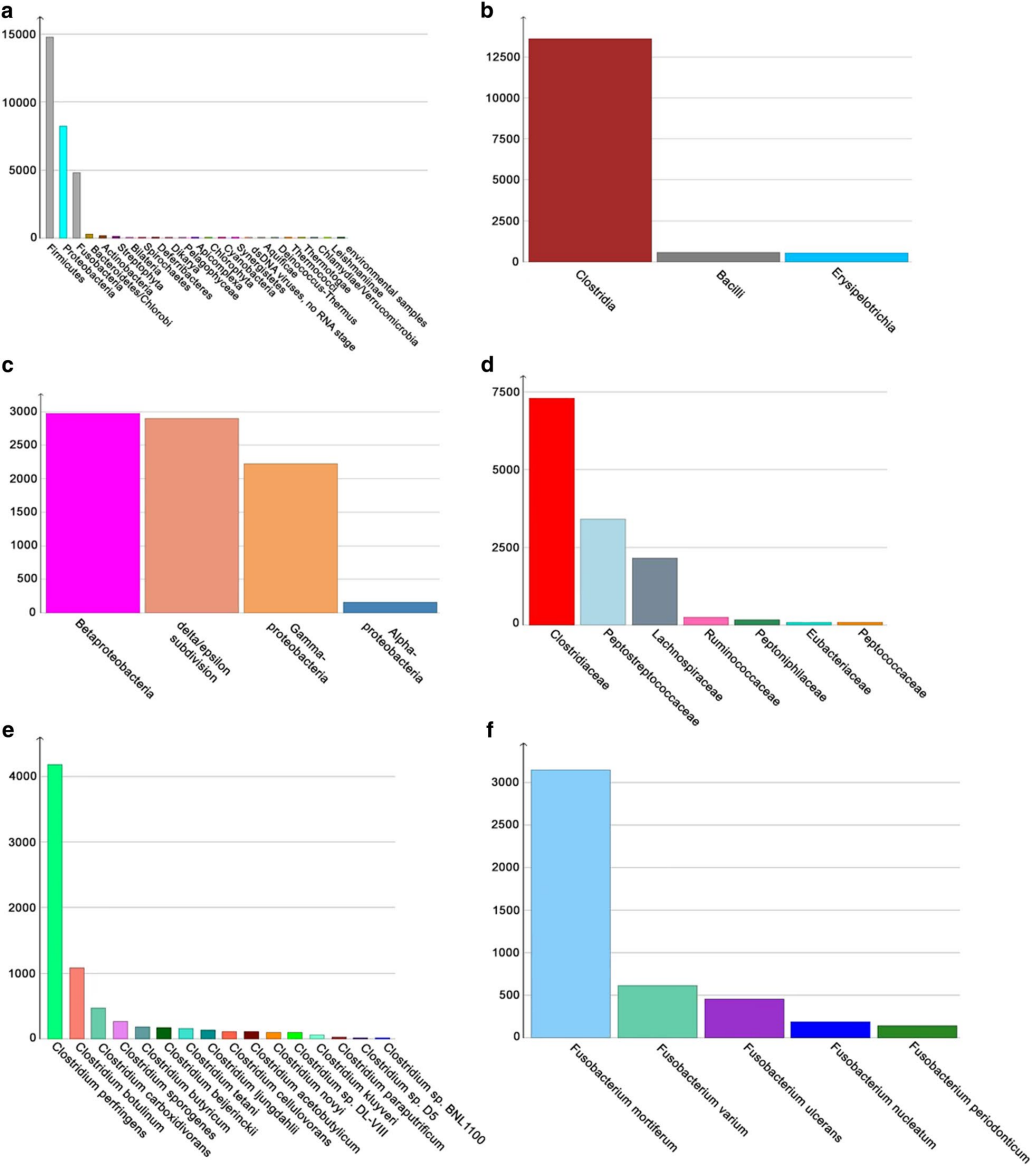
Taxonomic profile of the most abundant gut microbiota with MEGAN filtered NR gene catalogue. **a** Phylum level, **b** Firmicutes, **c** Proteobacteria, **d** Clostridiales, **e** *Clostridium*, and **f** *Fusobacterium*

From the mapping of the reads to the NR gene set catalogues obtained from MOCAT, the taxa of the top most abundant proteins in the facial skin microbiome were from *Sporidiobolales* (fungi), *Orthoretrovirinae* (virus), *Pleosporaceae* (fungi), *Bacillus cereus*, *Streptococcus* spp., and *Clostridiales*. While in the gut microbiome they were species from the genera *Bordetella*, *Mycobacterium*, *Chlamydia*, *Clostridium*, *Blautia* (a genus identified in the mammalian gut [50, 51]), and *Carnobacterium* (certain species inhibit the growth of *Listeria monocytogenes* in cured meats [52, 53]). The results from the search against Uniprot analysed with MEGAN (Figs. 1, 2) showed that in the facial skin microbiome the dominant population was *Proteobacteria,* followed by *Bacteroidetes*, *Firmicutes*, and *Actinobacteria*, with *Fusobacteria* in 12th place. Deeper examination of the *Proteobacteria* from the facial skin microbiome showed that the most abundant taxa were *Burkholderiales* from the *Betaproteobacteria*, and *Pseudomonadales* from the *Gammaproteobacteria* (Fig. 1b). From the *Pseudomonadales*, the most abundant taxon was *Psychrobacter* (mainly *P. cryohalolentis*, and *P. articus*), followed by *Pseudomonas* (mainly *P. stutzeri*, *P. aeruginosa*, and *P. putida*) (Fig. 1d–f). From the *Bacteroidetes*, the most abundant taxa were *Prevotellaceae* (mainly *P. ruminicola*) and *Flavobacteriaceae* (mainly from unclassified *Flavobacteriaceae* followed by *Flavobacterium*) (Fig. 1c).

*Firmicutes* was the most abundant phylum in the gut microbiome, followed by *Proteobacteria*, *Fusobacteria* in the third place, and *Bacteroidetes* in much less abundant in the fourth place. The most abundant class within *Firmicutes* was *Clostridia* (Fig. 2b). Among the *Clostridiales*, the most abundant families were *Clostridiaceae*, *Peptostreptococcaceae* and *Lachnospiraceae* (Fig. 2d). The most abundant taxa in the genus *Clostridium* were the potentially pathogenic *C. perfringens* and *C. botulinum*, followed by the beneficial *C. carboxidovorans*, *C. sporogenes*, and *C. butyricum* (Fig. 2e). The most abundant taxa from the *Proteobacteria* were *Burkholderiales* (from the *Betaproteobacteria)*, *Epsilonproteobacteria* (from the delta/epsilon subdivision), and *Enterobacteriales* (mainly from *Escherichia* from the *Gammaproteobacteria*) (Fig. 2c). The most abundant taxa in the *Fusobacteria* were the potentially pathogenic *Fusobacterium mortiferum*, *F. varium*, and *F. ulcerans* (Fig. 2f).

Many *Clostridia* that are part of the normal human gut microbiome were also found in the vulture gut, likely playing roles in digestion. For instance, *C. saccharolyticum* was significantly more abundant in the gut. This bacterium, which is present in sewage sludge, ferments various carbohydrates into acetic acid, hydrogen, carbon dioxide, and ethanol [54], functions for which we identified related genes in the vulture gut microbiome. We also identified genes for cellulose degradation in the gut, along with the cellulose degraders *C. cellulovorans* and *C. lentocellum* [55], which were significantly more abundant in the gut microbiome. The gut microbiome also contained *Bacteroides xylanisolvens*, which breaks down xylan [56] and for which we identified a gene related to this function. Also, significantly more abundant in the gut microbiome than in the facial skin were the butanol producers *C. beijerinckii* [57] and *C. saccharobutylicum* [58].

We also identified protein coding genes involved in vitamin biosynthesis in the gut strict MOCAT NR functional core. For example, from the genera *Hydrogenophaga*, *Herbaspirillum*, and *Gordonia,* we identified the genes for d-threo-aldose 1-dehydrogenase, involved in ascorbate and aldarate metabolism, cobalamin biosynthesis, riboflavin biosynthesis, and vitamin B_1_ biosynthesis; and 2-ketopantoate reductase, involved in vitamin B_5_ production [59]. Using the MGmapper gene core, which does not take taxa into account, we identified a larger abundance of genes in the gut than in the facial skin microbiome that belong to the metabolism of cofactors and vitamins (e.g. folate biosynthesis, vitamin B_6_ metabolism, riboflavin metabolism, and retinol metabolism). We also identified genes for the biosynthesis of various essential amino acids.

### Comparison of the facial skin and gut microbiome variation

**Fig. 3:**
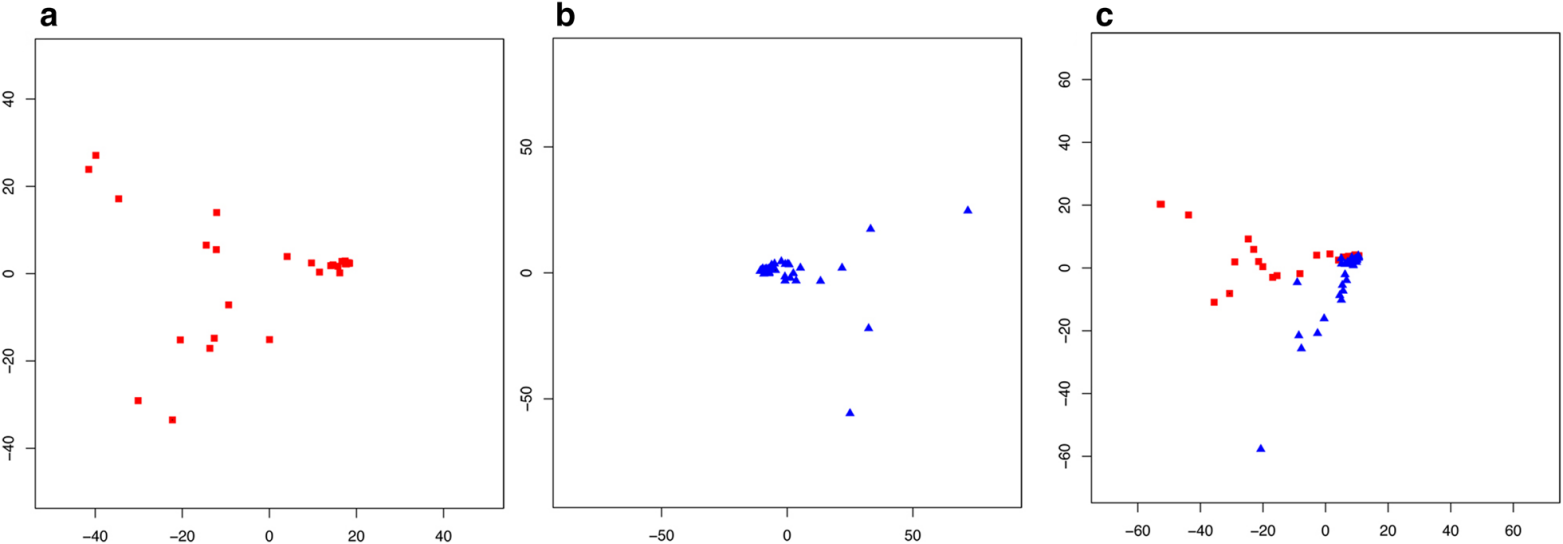
PC1 vs PC2 of the taxonomic species level abundance. **a** Facial skin microbiome, **b** gut microbiome, and **c** species present in both facial skin (red squares) and gut (blue triangles)

Based on the PCA of the abundance of the identified species in the facial skin and gut datasets (Fig. 3a, b), we found that the 803 species identified as driving the variation in the facial skin microbiome can be broadly grouped as: (i) pathogenic bacteria to a mammalian host (e.g. species from the genera *Bordetella*, *Gordonia*, *Shigella*, *Yersinia*, *Brucella*, *Prevotella*, and *Treponema*); (ii) soil or plant related; and (iii) related to mucosal surfaces and normal oral microbiome (*Neisseria* and *Nocardia*). Phages of *Salmonella*, *Aeromonas*, and *Erwinia* were also among the identifications driving variation. The 406 species not driving most of the variation included genera related to bioremediation (e.g. *Acinetobacter*), as well as other pathogens (species from the genera *Arcobacter* and *Brucella*), and phages of *Clostridium*, *Pseudomonas*, *Shigella*, and *Staphylococcus*.

In the gut samples, we identified 604 species that showed significant variation in abundance among the samples and 348 that were relatively uniformly distributed. The variation drivers included 112 potentially pathogenic bacteria, such as species from the genera *Listeria*, *Shigella*, *Yersinia*, *Bordetella*, *Shewanella*, *Erwinia*, and *Vibrio*. Bacteria that were non-drivers included species from the genera *Escherichia*, *Bacillus*, *Brucella*, and *Clostridium*, among others. Non-variation driver phages included phages for *Escherichia*, *Enterobacteria*, and *Shigella*. Phages driving variation included phages for *Clostridium*, *Yersinia*, and *Pseudomonas*.

Of the 879 microbial species shared by the facial skin and gut samples (Fig. 3c), we identified 553 species as driving variation (62.9%), and 326 as non-variation drivers (37.1%). Among the most important variation drivers were *Yersinia*, *Ralstonia*, *Rhizobium*, *Bifidobacterium*, *Bordetella*, *Listeria*, and *Burkholderia*. The non-variation drivers included *Brucella*, *Treponema*, *Clostridium*, and *Campylobacter*. Looking at the phages, only *Pseudomonas* phages were variation drivers, while non-variation drivers included phages for *Clostridium*, *Enterobacteria*, *Erwinia*, and *Shigella*.

### Functional potential characterization

**Fig. 4:**
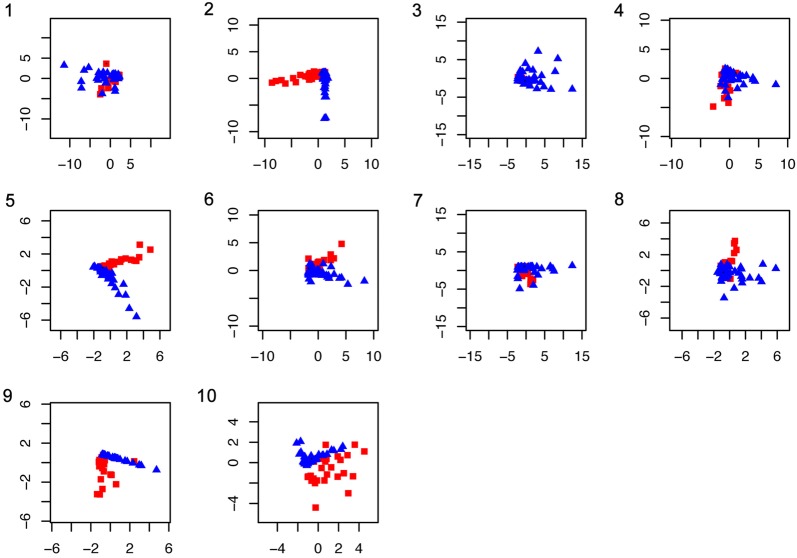
PC1 vs PC2 of the gene abundance from each pathway class. Red squares represent facial skin samples, blue triangles represent gut samples. The examined pathway classes were: **1**—Amino acid metabolism. **2**—Metabolism of other secondary metabolites. **3**—Carbohydrate metabolism. **4**—Energy metabolism. **5**—Glycan biosynthesis and metabolism. **6**—Lipid metabolism. **7**—Metabolism of co-factors and vitamins. **8**—Metabolism of other amino acids. **9**—Metabolism of terpenoids and polyketides.** 10**—Biodegradation and metabolism of xenobiotics

The PCA of gene abundance from the MOCAT NR gene set catalogue of the pooled facial skin and gut microbiomes annotated with KEGG showed less variation than the taxonomic profile in most of the pathway classes (Fig. 4).

**Fig. 5:**
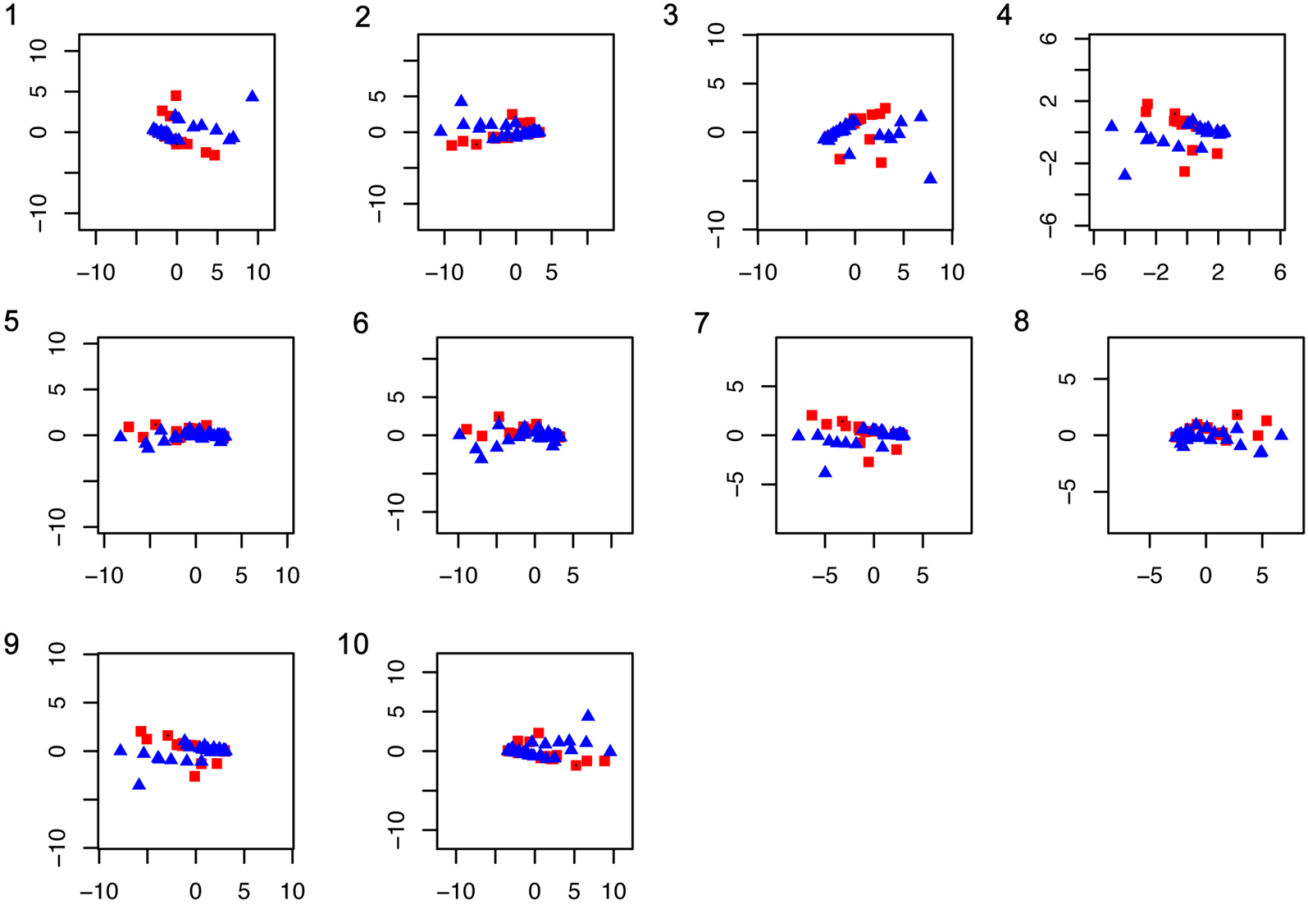
PC1 vs PC2 of each pathway class protein counts. Red squares represent facial skin samples, blue triangles represent gut samples. The examined pathway classes were: **1**—Amino acid metabolism. **2**—Metabolism of other secondary metabolites. **3**—Carbohydrate metabolism. **4**—Energy metabolism. **5**—Glycan biosynthesis and metabolism. **6**—Lipid metabolism. **7**—Metabolism of co-factors and vitamins. **8**—Metabolism of other amino acids. **9**—Metabolism of terpenoids and polyketides. **10**—Biodegradation and metabolism of xenobiotics

We also examined the number of proteins identified from each pathway class in the MGmapper NR protein set. PC1 of each pathway class explained 78–99% of the variance and clearly separated the pathway classes by their functional composition (Fig. 5). Examination of the rotation matrix showed that 60.4% (87) of the sub-pathways were responsible for the observed variation between the facial skin and gut microbiomes. Analysing variation by sample type, 57% of the sub-pathways in the facial skin (81 sub-pathways) and 56% (80 sub-pathways) in the gut datasets drive intra sample variation. We identified 59 variation driver genes with the largest (top 5%) mean abundance difference between facial skin and gut. Of those genes, 18 corresponded to amino acid metabolism (17 only present in gut samples and one in a single facial skin sample), 15 genes from carbohydrate metabolism (all in gut samples), and 7 genes corresponding to metabolism of cofactors and vitamins (all in gut samples). We found that 44% of the genes (921 out of the KEGG annotated 2093 pooled facial skin and gut relaxed gene cores) did not drive variation. The 46 genes with the smallest (bottom 5%) mean abundance difference between facial skin and gut were associated to the metabolism of amino acids, carbohydrates, cofactors and vitamins, glycan biosynthesis and metabolism, lipid metabolism, energy metabolism, chlorocyclohexane and chlorobenzene degradation, metabolism of terpenoids and polyketides, and metabolism of other amino acids.

Euclidean distance measures among the gut samples showed less intra sample type variation than the facial skin samples (Table 1, P = 0.002). The facial skin microbiome distances ranged from 3.2 to 6.2, while those in the gut microbiome ranged from 2.2 to 3.7. Analysis of all the functions together instead of per pathway class showed that the facial skin and gut microbiomes clearly separated into two different clusters. The MOCAT functional characterization yielded a total of 38,403 NR genes from the facial skin dataset and 50,106 NR genes from the gut dataset. Based on the normalized abundance of the mapping reads, we identified 1507 genes in the facial skin strict core and 7215 in the relaxed core, and 157 top abundant genes. We found 2512 genes in the gut strict core, 14,028 in the relaxed core, and 151 top abundant genes.

**Table 1:** Distances between and within the facial skin and gut samples

Pathway	Facial skin vs facial skin	Gut vs facial skin	Gut vs gut
Amino acid metabolism	4.87	4.66	2.86
Biosynthesis of other secondary metabolites	5.97	5.97	3.68
Carbohydrate metabolism	4.1	4.48	3.58
Energy metabolism	3.33	3.27	2.17
Glycan biosynthesis and metabolism	3.24	3.67	3.22
Lipid metabolism	5.6	5.66	3.03
Metabolism of cofactors and vitamins	4.27	4.41	2.99
Metabolism of other amino acids	3.48	3.77	2.75
Metabolism of terpenoids and polyketides	4.57	4.94	3.18
Nucleotide metabolism	1.50	1.56	1.34
Xenobiotic biodegradation and metabolism	6.22	5.98	3.61

### Core microbiome identification and attributes comparison

In the filtered MGmapper taxonomic profiling, we identified 1483 species in the facial skin samples, 638 of which occurred in the relaxed core, and only 184 species that occurred in the strict core. In the gut microbiome, we found 1419 microbial species, with 322 present in the relaxed core, and 129 in the strict core. In the functional characterization, we identified a total of 238,065 NR unique bacterial genes in the facial skin microbiome and 387,951 NR unique bacterial genes in the gut microbiome (Additional file 3).

We compared the taxonomic and functional composition of the facial skin and gut microbiomes and examined the microbial attributes of the taxa identified from the annotations of the assembled genes (Additional file 3). We found ~ 26× more habitat-specialized microbes in the facial skin than in the gut microbiome (facial skin = 8373, gut = 320). Consistent with the anaerobic gut environment, the gut microbiome had ~ 5× more anaerobic or microaerophilic bacteria than the facial microbiome (gut = 34,749, facial skin = 6699). The functional pathways clearly separated by sample type were the metabolism of other secondary metabolites, the glycan biosynthesis and metabolism, and the lipid metabolism, followed to a lesser extent by the metabolism of xenobiotics and the metabolism of other amino acids (Fig. 4). From the energy metabolism class, the methane metabolism was among the most abundant functions in the facial skin and gut microbiomes.

### Pathogenic characterization

There was no significant difference (P = 0.44) in the number of identified potentially pathogenic plasmids between the facial skin (x̅ = 11.3) and gut (x̅ = 9.82) microbiomes. Likewise, there was no statistical difference in their abundance between the facial skin and gut samples (P = 0.78). Furthermore, no potentially pathogenic plasmid was present in 90% of the samples. Among those present in at least 50% of the samples were plasmids from *Burkholderia vietnamiensis*, *Escherichia coli*, and *Ochrobactrum anthropic*. Potentially pathogenic plasmids present only in the facial skin microbiome were from opportunistic pathogens such as *Acinetobacter baumannii* and *Staphylococcus epidermidis*, which is usually part of the normal skin microbiome [60]. For example, the *O. anthropi* ATCC 49188 plasmid pOANT04 was present in 63 of the samples. *O. anthropi* is being increasingly recognized as a potentially problematic opportunistic and nosocomial human pathogen [61].

The Shiga toxin 1-converting phage BP-4795, which transmits virulence genes to its infected bacteria [62], was found in only five facial skin samples, and in 23 of the gut samples at various levels of abundance (max. coverage = 17.9%, max. mapping reads = 372). We also found the *Shigella* phage SfIV, which aids the virulence of *Shigella flexneri* [63], in 25 of the gut samples and 8 of the facial skin samples. We found that 75.2% of the identified pathogens are classified as level 2 pathogens in the facial skin samples (level 1 = 26 species, level 2 = 79 species), while 95.8% of those more abundant in the gut were classified as level 2 (level 1 = 2 species, level 2 = 46 species).

From the MGmapper strict core, the only protein identified in most of the facial skin samples (20 samples) and gut samples (36 samples) was an uncharacterized protein from *Chlamydophila psittaci*, an avian pathogen that causes avian chlamydiosis and epizootic outbreaks in mammals [64]. Among the viruses from this functional strict core, we found the avian endogenous retrovirus EAV-HP and the avian leucosis virus. We also identified in higher abundance in the gut dataset *Trichuris trichiura*, causative of trichuriasis in humans [65, 66] (max. mapping reads_facial skin_ = 942, max. mapping reads_gut_ = 565,870), and *Eimeria brunetti*, causative of haemorrhagic intestinal coccidiosis in poultry [67] (max. mapping reads_facial skin_ = 30, max. mapping reads_gut_ = 1706). More abundant in the facial skin dataset we identified the fly *Lucila cuprina* (x̅_gut_ = 11,910, x̅_facial skin_ = 49,210), which causes sheep strike [68].

We identified only 49 bacteria in the gut dataset with potential for sporulation, including *Fusobacterium necrophorum*, *Campylobacter jejuni*, and *L. monocytogenes* (Additional file 4). Regarding the bacteria reported as zoonotic pathogens, we could only identify *Streptococcus suis*, a pathogen capable of transmission from swine to humans [69]. Other identified bacteria with reported zoonotic capacity had very low abundance and were present in only one or two samples, so that they likely represent non-viable bacteria already dealt with by the vulture.

## Discussion

### Microbiome composition and variability

**Fig. 6:**
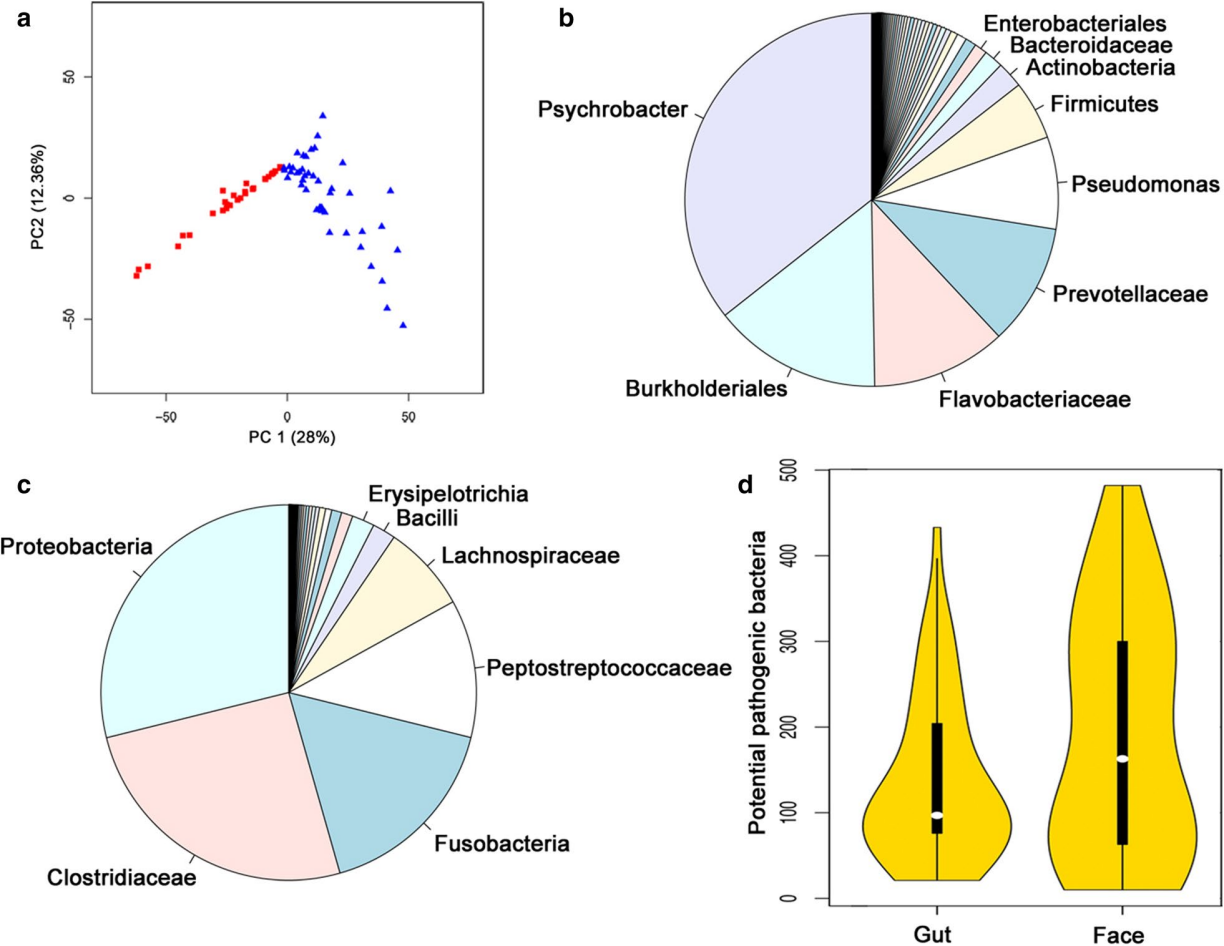
Vulture facial skin and gut microbiome composition. **a** Principal component (PC) 1 (28% of the variation) and PC 2 (12.4%) of the abundance of all genes from all the KEGG metabolic classes together of the facial skin (red squares) and gut (blue triangles) samples. **b** Taxonomic profile of the facial skin and **c** gut microbiota. **d** Distribution of the number of identified potentially pathogenic bacteria in the gut and facial skin datasets

The comparison of our metagenomic bacterial identifications to those by Roggenbuck et al. [17] confirm the consistency of the taxonomic identifications. Given that we aim at characterizing the vulture scavenging-related microbiome, in light of previous observations that the facial skin and gut microbiota of turkey and black vultures largely overlap [17], we combined the datasets of both vulture species into one. The results from comparing the number and relative abundance of identified taxa in the two vulture species prove that their microbiomes are not statistically different (P = 0.68), and validate their joint use. We identified a strikingly large taxonomic and functional variation within the gut and facial skin datasets (Figs. 3, 4 and 6a). The observation that the functional profiles showed less variation than the taxonomic profiles could suggest a large amount of functional redundancy in the microbiota, or that there is a need for a common set of functions in order to thrive on the vulture’s facial skin and gut. Examination of the PCA from the functional potential characterization suggests that the functional profile of the facial skin and gut microbiomes are very similar in most of the pathway classes, despite large within-sample type variation (Fig. 4). This suggestion is supported by the PCA of the number of identified proteins belonging to each pathway class (Fig. 5). This analysis further suggests that the relative abundance of the proteins rather than the presence/absence of them is one of the main factors distinguishing the facial skin from the gut microbiome functional profile. In comparison to the facial skin microbiome, the gut had less variation in the functional profile (Table 1, P = 0.002). This is consistent with the fact that the facial skin is the first part of the vulture’s body to make contact with the carcass, thus potentially becoming contaminated by the carcass, which would lead to large variation in the facial skin microbiota than the more ecologically constrained gut microbiota. In general, the identified taxa from the gut and facial skin microbiomes can be hypothesized to derive from (i) host, such as *Methanobrevibacter smithii* in the gut, and (ii) environment and carcass, e.g. *Xanthomonas* and *Actinobacillus pleuropneumoniae*. Determination of the carcass microbiota would be necessary for further evaluation of this grouping; however, it was not possible to obtain samples of the carcass the sampled vultures were feeding from. Given that the most abundant facial skin microbes can be associated to a variety of microbial attributes ranging from producers of antifungals to usual intestinal bacteria and plant and soil related bacteria, it is clear that there is a large environmental and carcass microbiota input to the vulture’s highly variable facial skin microbiome. On the other hand, those most abundant in the gut were related mainly to intestinal or faecal bacteria, reflecting the digestive and more specialized functions expected to occur in the gut. Our data confirms previous PCR-based results [17] that identify *Clostridia* and *Fusobacteria* as dominant taxa in the gut microbiome (Figs. 2, 6c). As expected, we identified clear traits (bacterial taxa and genes) in the gut microbiome for carrying out digestive and nutritional activities.

### Reduced core host microbiome

In order to differentiate the constant host microbiome from the one derived from variable and external influences (i.e. microbes derived from the carcass and the environment), we defined two types of microbiome cores. A relaxed core containing those elements (microbial species and genes) present in at least 50% of the samples, and a strict core with those present in at least 80% of the samples. We found that the relaxed core contained ~ 43% and ~ 22.7% of the facial skin and gut taxa, respectively, and the strict core only contained ~ 1% of the taxa in both gut and facial skin datasets. Notably, the distinction between carcass and established constant host-derived microbiome was complicated, even after the cores were defined. For example, the foodborne pathogen *Salmonella enterica* was present in the gut core (Fig. 6b). We additionally identified genes in the facial skin and gut microbiome functional strict cores that are related to putrescine, one of the main molecules produced in a carcass (Additional files 5 and 6). We found ~ 26× more habitat-specialized microbes in the facial skin than in the gut microbiome (facial skin = 8373, gut = 320), most likely due to the fact that a mammalian corpse is a disturbance habitat that selects for a specialized microbial community [13]. Some of these community species likely derive from the carrion microbiota. For example, we identified phenol degrading bacteria in the vulture facial skin, such as *Acinetobacter calcoaceticus* [70] (max. mapping reads_facial skin_ = 478, max. mapping reads_gut_ = 6), for which we identified its gene coding for phenol 2-monooxygenase in the NR gene catalogue. Phenolic compounds can act against foodborne pathogens and spoilage bacteria [71], suggesting that they derive from carrion dwellers, adapted to their competitive environment, instead of being part of the core vulture facial skin microbiome. Thus, we suggest that the vulture microbiome is a result of its scavenging diet, with part of the carcass microbiome leaving a profound footprint in the vulture microbiome.

### Pathogenicity challenges

**Fig. 7:**
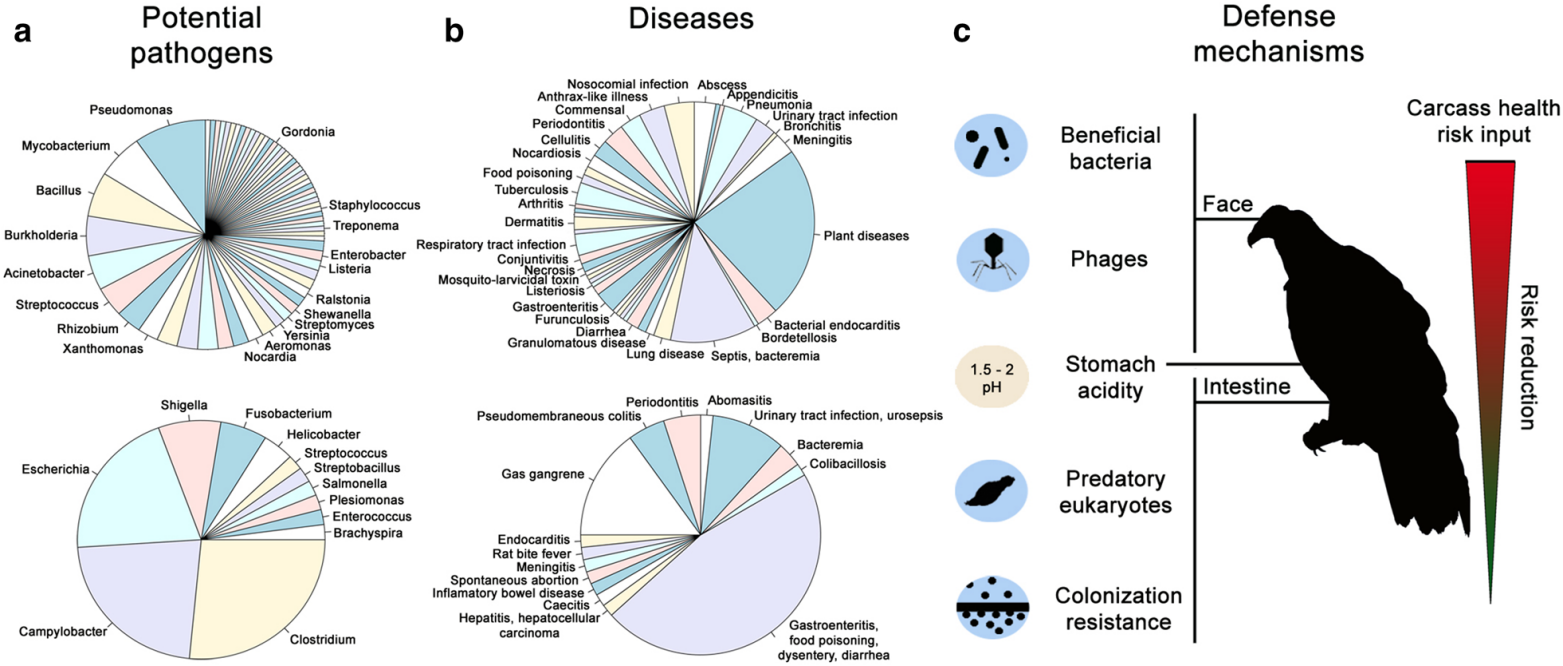
Health challenges faced by the vulture. **a** Vultures are confronted with a wide variety of carcass-derived microbes (**b**) that pose serious pathogenic risks to non-scavenging species. **c** Different potential microbiome-mediated defence mechanisms account for the vulture’s ability to tolerate and reduce the health-risk potential that a carcass represents as it passes through its digestive system

Given the identification of a strong carcass microbiome signature in the vulture microbiome, we next characterized all potential pathogens dealt with by the vultures. We defined potential pathogens as taxa and functions that, while present in the vulture microbiome without conferring an apparently negative health effect, could be deadly for non-scavengers. Most of the significantly more abundant potential pathogens found in the facial skin microbiome (P < 0.05) are known to produce anthrax-like illnesses, periodontitis, pneumonia, and tuberculosis in mammals, while those found more abundantly in the gut are known to cause gastroenteritis, gas gangrene, food poisoning, and dysentery in humans (Figs. 6d, 7a, b). We identified several pathogenic plasmids in the gut microbiome, such as the Shiga toxin 1-converting phage BP-4795, which transmits virulence genes to the infected *E. coli* [62], as well as genes in the facial skin microbiome related to pathogenicity, such as haemolysins (Additional file 6). Our untargeted metagenomics approach also identified non-bacterial potential pathogens in the gut, such as the round worm *T. trichiura*, causing trichuriasis in humans [66], and the apicomplexan parasite *E. brunetti*, responsible for haemorrhagic intestinal coccidiosis in poultry [67]. These results highlight the health-challenging environment dealt by the vulture due to its scavenging diet.

### Stomach acidity protection

The vulture stomach acidity has been suggested to prevent potential pathogens from reaching the gut [14, 17]. However, postprandial pH values observed in the stomach of black and turkey vultures appear to be no more acidic than those reported for domestic fowl and non-scavenging birds that consume large animal prey [72]. Instrument readings of the pH are usually higher in a stomach with food contents, since the gastric acid is sparse and diluted by the water content in the lumpy food items. Sequential and independent probe values in the stomach can often provide different readings, even when the probe is used in the same location. This is reflected in the large standard deviations observed in the previously reported pH readings of black vultures (pH 3.8 ± 1.25) [72]. The measurements were less acidic, and neutral in some occasions in the duodenum (pH 6.1 ± 0.48) and lower intestine (pH 6.0 ± 0.3) [72]. Given that the carcass microbiome enters the vulture’s body mainly along with the ingested food items, the pH measurements suggest that the gastrointestinal acidity is not an efficient filter against all the potential pathogens present in a scavenging diet, rather it plays the general role of primary selection, which is not enough for all the potential pathogens in the carcass.

Most of the potential pathogens identified were restricted to few samples, and the abundance of the pathogens was not consistent across samples after count normalization. These observations could be due to variation in the carcass microbiome, or efficient elimination of the potential pathogens by the vulture. In the comparison of the facial skin and gut microbiomes, we found that the facial skin had more species of potential pathogens than the gut (P = 0.036) (Fig. 6d, Table 2). However, there was no statistical difference in their abundance (P = 0.82), and the gut still harboured various potential pathogens. Interestingly, among the non-bacterial identifications we found the Chinese liver fluke, *Clonorchis sinensis* in 22 of the facial skin samples and 39 of the gut samples (66.6% of the facial skin and 83% of the gut samples). This liver fluke feeds on bile and causes problems in fat digestion, and it is able to reach the gut of the hosts given its acidity resistance [73].

**Table 2:** Top 10 potential disease-causing bacteria identified in the facial skin and gut

Pathogen	Disease	x̅ facial skin	Rank facial skin	x̅ gut	Rank gut
*Stenotrophomonas maltophilia* K279a	Bacteraemia, bronchitis, pneumonia, urinary tract infection	75,292	1	18,298	7
*Clostridium perfringens* ATCC 13124	Gas gangrene	61,879	2	166,694.6	1
*Plesiomonas shigelloides* 302-73	Gastroenteritis	48,642	3	134,119	2
*Clostridium perfringens* str. 13	Gas gangrene	41,308	4	125,717.5	3
*Pseudomonas fluorescens* A506	Commensal (plant)	31,324.9	5	271.2	49
*Clostridium perfringens* SM101	Gas gangrene	30,361.2	6	109,973.2	4
*Acinetobacter lwoffii* SH145	Nosocomial infections	19,950.2	7	3.2	149
*Aeromonas salmonicida* subsp. salmonicida A449	Furunculosis	14,347.4	8	45.4	92
*Acidovorax avenae* subsp. avenae ATCC 19860	Bacterial leaf blight, brown stripe, red stripe	13,111.2	9	998.7	30
*Propionibacterium propionicum* F0230a	Commensal	11,896.8	10	73.64	80
*Campylobacter lari* RM2100	Gastroenteritis, diarrhoea	4791	26	53,435	5
*Campylobacter jejuni* subsp. doylei 269.97	Bacteraemia	477.2	108	24,733.9	6
*Campylobacter jejuni* RM1221	Food poisoning	327.4	120	13,136.9	9
*Fusobacterium nucleatum* subsp. nucleatum ATCC 25586	Periodontitis	2166	57	13,456.6	8
*Clostridium perfringens* E str. JGS1987	Gastroenteritis, gas gangrene	3398.2	39	12,724.7	10

### Microbiome mediated protection

It has been shown that microbes provide protection to the host against pathogenic bacteria, thus we hypothesized that the vulture microbiome plays a protective role in terms of combating, preventing or maintaining in balance the abundance of potential pathogens. Accordingly, we identified functional and taxonomic protective elements that could be classified as related to (i) beneficial bacterial taxa and functions, (ii) phages, (iii) predatory eukaryotes, and (iv) colonization resistance (Fig. 7c, Additional file 5).

#### Beneficial bacterial taxa

Consistent with our microbiome-mediated protection hypothesis, we identified *Hylemonella gracilis* as part of the facial skin core, which has been shown to prevent long term colonization by *Yersinia pestis* [74]. Other beneficial bacteria present in both gut and facial skin microbiomes include *Lactobacillus sakei*, an antilisterial bacterium [75]. We also identified several genes for the biosynthesis of antibiotics such as carbapenem, tetracycline, macrolides, and ansamycins, as well as resistance genes towards them (Additional file 6). The identification of insecticide, fungicide, and antiparasite related taxa and genes in the facial skin microbiome suggests protective mechanisms against possible eukaryotic pathogens present in the carcass (Additional file 6). For example, *Pseudomonas entomophila,* which causes lethality in flies [76], and for which we identified a gene coding for an insecticidal toxin SepC/Tcc class in the facial skin NR gene set catalogue (Additional file 5). The production of antibiotics to outcompete for resources is known in soil microbiomes, and recently similar strategies have been reported in the human nasal microbiome from commensal bacteria against pathogens [77]. Our results suggest that the vulture’s facial skin microbiome plays a similar defensive role. Regarding the gut microbiome, commensal *Clostridia* are known to play an important role in the production of butyrate that the colonocytes use [78]. Notably, *C. butyricum* was among the most abundant *Clostridia* in the gut microbiome. Future studies would be needed to go beyond the description presented here to test for competitive exclusion among bacteria in the vulture gut microbiome [79, 80].

#### Beneficial bacterial functions

Besides containing potential pathogenic microbes, carcasses also contain toxic and carcinogenic compounds [81], which pose health risks to the vulture, particularly to its facial skin, which is in direct contact with such compounds. Among the bacteria identified in higher abundance in the facial microbiome was *Arthrobacter phenanthrenivorans*, which is able to degrade phenanthrene, a skin-irritating polycyclic aromatic hydrocarbon (PAH). PAH are xenobiotic pollutants with negative health-effects found to be emitted from animal carcass [82], and previously reported in high concentrations in other vulture species [83]. Interestingly, the largest variation on the metabolism of xenobiotics biodegradation was in the facial skin dataset (Additional file 6), with PAHs degradation metabolism being the most abundant subclass from the xenobiotics degradation pathway in both facial skin and gut. These findings suggest a microbiome protective role for the vulture against such compounds. In regards to the gut microbiome, the second most abundant *Fusobacteria* in the gut from the NR gene set was the gut butyrate-producing *F. varium* [84], for which we also identified its gene formate C-acetyltransferase, which is involved in butanoate metabolism, as the most abundant gene in the gut dataset. Interestingly, besides the use of butyrate for the colonocytes, it has been shown that butyrate glycerides have antimicrobial activity against *C. perfringens* and *Salmonella typhimurium* [85].

#### Phage controlled pathogen abundance

Phages in the human gut microbiome have been shown to play a protective role and the increasing identification of antibiotic resistance genes in pathogenic bacteria has led to the proposition of using phages as alternative therapies [86]. Given the identification of potential antibiotic resistance genes in the vulture facial skin and gut microbiomes (Additional file 5), we investigated the possible role of phages in eliminating or balancing the abundance of potential pathogens. In the facial skin microbiome, *Clostridium* phages positively correlated with *C. perfringens* and *C. botulinum*, whereas in the gut we observed enterobacteria phages correlating to *Escherichia fergusonii* (Additional file 7). Furthermore, in the taxonomic annotations of the gut functional core, we identified the *Salmonella* phage SPN3US (Additional file 7), which has shown effective inhibition of *S. enterica* [87]. From the facial skin functional strict core, the most abundant virus was phage BPP-1 (Additional file 7), which infects pathogenic *Bordetella* bacteria [88]. These findings show that the phage sets in both facial skin and gut microbiomes are related to the presence of the potential pathogens most abundant in the corresponding sample type. They also suggest that phages could represent an alternative defence mechanism for the control, and possibly elimination of potential pathogens, as in phage therapy [89] (Additional file 5).

#### Predatory defence mechanism

In spite of being important elements in the gut microbiome, gut microbial eukaryotes remain largely unexplored. Thus, we investigated whether vulture gut microbial eukaryotes played any protective role. We identified the invertebrate *Adineta vaga,* which feeds on dead bacteria and protozoans, to be ~ 6.8× more abundant in the gut core than in the facial skin core. This identification suggests that a predatory mechanism may be exploited for defence in the vulture’s gut.

#### Biofilm formation and colonization resistance

Biofilms are assemblages of microbes associated within a matrix composed of extracellular polymeric substances that facilitate their adhesion to the surface, protection against antimicrobials, and better nutrient acquisition. The abundance of *Fusobacteria* in the gut has been suggested to play a particularly relevant role in lumen biofilm formation in the gastrointestinal tract [90]. To explore this hypothesis, we searched for proteins related to biofilm formation (Additional file 7). In the core of the gut functional potential we identified biofilm related proteins from *F. mortiferum*, such as rubrerythrin, as well as from *C. perfringens*, such as UDP-glucuronic acid epimerase. Given that bacteria form biofilms in which they can thrive under different patterns of gene expression [91], this suggests that the identified potential pathogenic *Clostridia* and *Fusobacteria* from the gut microbiome might not pose pathogenic risks and instead offer colonization resistance against other external pathogens (Additional file 5). To further explore the possible colonization resistance role of *Clostridia* and *Fusobacteria*, we examined the gut functional core for toxins with potential effects on the vulture. We identified only few potentially pathogenic toxin coding genes from *Fusobacterium* (Additional file 7). Considering that *F. varium* has been shown to affect its human host in a beneficial manner by antagonizing colonization by pathogenic agents [92], we suggest that an important role of the gut *Fusobacteria* could be the formation of biofilms and colonization resistance, without representing a serious pathogenic threat. We identified the pathogenicity genes perfringolysin O and phospholipase C in the gut microbiome from *C. perfringens*. However, we also identified genes for the biosynthesis of short chain fatty acids from *Clostridia*, which can provide protection against inflammatory responses [93]. Thus, we could classify the observed *Clostridia* into two types, (i) the potentially pathogenic, mainly represented by *C. perfringens* and (ii) the non-pathogenic, which may contribute to biofilm formation and health defence (Additional file 4).

## Conclusions

Our findings strongly suggest that the turkey and black vultures have adapted to their scavenging diet with the help of their facial skin and gut microbiomes. Surprisingly, most of their microbiome consists of a large variable pool of environmental and carcass-derived microbiota, with only a small set of constant inhabitants. In particular, the presence of a wide variety of microbes reported as pathogenic to non-scavengers (mammals and other birds) without an apparent or a reported pathogenic effect on the vultures calls for deeper study. Further studies would be required to determine whether the microbes reported here are pathogenic to the wild vultures or if they serve as reservoirs, and to determine what is their zoonotic potential. A better characterization of wild-life as potential pathogenic reservoirs (with microbiological, epidemiological and surveillance data) would allow for better informed wild-life protection programs, particularly for those species in endangered status, such as some species of vultures (e.g. the white-backed vulture [94]). We highlight the identification within the vultures’ facial skin and gut microbiomes of defence mechanisms that are alternative to the use of antimicrobials, such as the use of predatory microbes, and the protective nature of colonization resistance through biofilm formation by Fusobacteria and Clostridia. However, further microbiology studies would be needed to isolate the relevant microbes and validate the antimicrobial mechanisms reported here from the vultures’ microbiomes.

The establishment of these suggested protective mechanisms in the vulture microbiome unveiled by metagenomics analyses highlights the important role that vultures play in their ecosystem. This role is the essential but underrated service of cleaning up carcasses that otherwise would spread microbial elements pathogenic to species without a specialized microbiome like that of the vultures. In conclusion, our results show the importance of complementing genomic analyses with metagenomics on the host microbiome in order to obtain a clearer understanding of the host-microbial alliance that aids the evolution of extreme dietary adaptations.

## Additional files

**Additional file 1.** The number of sequencing reads at the different filtering stages and metadata information on the samples (sheet 1). It also contains the read mapping counts to the various whole genome databases with MGmapper using the facial skin (sheet 2) and gut (sheet 3) datasets, as well as the bacterial taxonomic identifications from the 16S results in the study by Roggenbuck et al. [17] not found in the metagenomic bacterial taxonomic identification (sheet 4).

**Additional file 2.** The identified differentially abundant bacteria of the facial skin microbiome compared to the gut microbiome using a t-test (sheet 1) and a Wilcoxon test (sheet 2). It also contains a summary of the taxonomic identifications from the whole-genomic databases (sheet 3) and a summary of the abundance of the identified differentially abundant species (sheet 4).

**Additional file 3.** The comparative results of the facial skin and gut microbiome taxonomic content (sheet 1) and microbial attributes (sheet 2) identified with MEGAN. It also contains stats on the facial skin and gut microbiome functional annotation (sheet 3) and the bacterial total genes from each pathway from KEGG (sheet 4).

**Additional file 4.** Identified potential pathogens in the vultures’ facial skin and gut microbiomes (sheet 1–4). The document also contains identified Clostridia toxin/antitoxins (sheet 5), pathogenic genes (sheet 6), strains with sporulation potential (sheet 7), and short chain fatty acids (sheet 8).

**Additional file 5.** Extra results and discussions on the vultures’ facial skin and gut microbiomes.

**Additional file 6.** The identified proteins related to xenobiotics metabolism from the facial skin and gut microbiomes (sheets 1 and 2), as well as the identified genes from the facial skin and gut microbiomes related to putrescine and other carcass compounds (sheets 3 and 4). It also contains the identified resistance genes (sheets 5 and 6), and information on other identified genes related to pathogenicity (e.g. haemolysins) and health protection (e.g. antiparasitics) (sheet 7).

**Additional file 7.** The correlation values of the microbes from the facial skin (sheet 1) and gut (sheet 2) microbiomes. It also contains supporting information on identified phages (sheet 3), biofilm formation genes (sheet 4), and *E. coli* toxin/anti-toxin genes (sheet 5), as well as the identified toxins from Fusobacteria (sheet 6) and Clostridia (sheet 7).
