# Supplementary material for: Protective role of the vulture facial skin and gut microbiomes aid adaptation to scavenging
Source: Acta Vet Scand. 2018 Oct 11;60:61. doi: 10.1186/s13028-018-0415-3 (PMC6182802; doi:10.1186/s13028-018-0415-3)
Supplement: Supplementary file 5 — Additional file 5. Extra results and discussions on the vultures’ facial skin and gut microbiomes. [file 13028_2018_415_MOESM5_ESM.pdf]

**Additional File 5**

This additional file contains extra results and discussions on the vultures' facial skin and gut microbiomes.

## Functional characterization

### *Facial and gut microbiomes comparisons*

We identified 2 genes from lipid metabolism, 2 genes from glycan biosynthesis, and metabolism and peptidoglycan biosynthesis, and 1 gene present only in the facial skin dataset related to phenylpropanoid biosynthesis from *Alistipes* (related to protection from UV light and defence against herbivores and pathogens [1–3]). Interestingly, ~98x more bacteria in the facial skin microbiome is annotated as moderate halophilic (facial skin= 11,239 taxa, gut= 115 taxa), as well as ~239x more psychrophilic bacteria in the facial skin (facial skin= 18,886 taxa, gut= 79 taxa). The top 5% bacteria with the largest difference in abundance from the pathways that drive variation between the facial skin and gut microbiomes, contains the metabolism of fructose and mannose, starch and sucrose, galactose, and amino sugar and nucleotide sugar, all of them more abundant in the gut microbiome.

### *Cadaverine and putrescine*

Three of the main molecules produced in a decomposing body are nitrate reductase (converting nitrite to ammonia) [4], cadaverine (lysine decarboxylase) [5], and putrescine (ornithine decarboxylase) [5]. In this regard, we identified in the facial skin MOCAT NR strict core a spermidine synthase gene from *Janthinobacterium* sp. HH01, and spermidine/putrescine ABC transporter ATPase from *Herbaspirillum* sp. GW103 in both facial skin and gut MOCAT strict cores. Sulphur compounds are also emitted by decomposing carcasses [6], likely derived from

methionine and cysteine degradation. This likely explains the identification of the metabolism of cysteine and methionine as the two most abundant subclasses from the amino acids metabolism in both facial skin and gut microbiomes. A carcass also produces volatile organic compounds [7], such as acetone, methyl ethyl ketone, toluene, ethylbenzene, m,p-xylene, styrene, and o-xylene. In this regard, toluene degradation was one of the subclasses not driving variation in the facial skin functional intra samples comparison, and the MOCAT facial skin cores had more genes related to xenobiotics biodegradation metabolism than those of the gut microbiome (Additional file 3).

## Digestive role of the gut microbiome

### *Intestinal microbiome related to digestion*

Among the taxa present in greater abundance in the facial skin microbiome than in the gut microbiome, we identified taxa and functions that are usually part of the gut microbiome of mammals. These bacteria could be derived from the carrion but be removed from the vulture gut microbiome. For example, present only in the facial skin dataset was *Cellulophaga lytica*, which is capable of degrading proteins and polysaccharides [8], as well as *Flavobacterium columnare*, which produces gelatin-degrading and chondroitin sulfate-degrading enzymes [9,10]. This is relevant given that chondroitin sulfate is one of the main structural components of cartilage. Although we did not identify these genes from *F. columnare*, we identified chondroitin sulfate ABC lyase genes in both facial skin (4 genes from *Bacteroides* spp. and *Proteus* spp.) and gut microbiomes (13 genes from *Bacteroides* spp., *Edwardsiella* spp., and *Proteus* spp.).

### **Fusobacterium** *digestive roles*

It has been proposed that the abundance of *Fusobacterium* in the gut could aid in the digestion of meat, given their ability to metabolize amino acids [11,12]. This suggestion is supported by the

finding of *F. nucleatum* and *F. varium* in the vulture's gut microbiome. One of the most abundant genes in the gut microbiome codes for an alpha-2-macroglobulin family protein from *F. mortiferum* (the most abundant *Fusobacterium* in the gut). This protein has been suggested to be used in bacteria as a colonization rather than a virulence factor [13]. Besides, eukaryotic alpha-2-macroglobulin, produced by the liver, binds to and removes MMP-2 and MMP-9 (active forms of the gelatinase), which is produced in the stomach to digest gelatin [13,14]. However, the gelatin colloidal properties aid in the digestion of various types of food [15,16]. Furthermore, bacterial alpha-2-macroglobulin can be structurally very similar to that of eukaryotes [17]. This suggests that *Fusobacterium* could also be playing digestive aiding roles in the vulture gut.

## Taxonomic characterization

### *Pathogenic characterization*

Looking at the identified bacteria taking into account the strain information, the maximum number of potentially pathogenic bacteria identified in a sample (a facial skin sample) was 482, and the minimum was 10, with $\overline{x}$= 159.8. Each pathogen was present with $\overline{x}$= 11.98, a minimum of 1, and a maximum of 75 (*Clostridium perfringens* ATCC 13124 and *C. perfringens* str. 13). We found that the facial skin has more different species of potential pathogens than the gut (*P*= 0.036, $\overline{x}_{facial\ skin}$ = 189.8, $\overline{x}_{gut}$ = 137.3). Present in at least 90% of the samples are three *C. perfringens* strains which produce gas gangrene [18], and one *Stenotrophomonas maltophilia*, which produces bacteraemia, bronchitis, pneumonia, and urinary tract infection [19]. In the gut samples, the most abundant hosts for the potentially pathogenic bacteria are human, chicken, turkey, cattle, pigs, and mouse. For those in the facial skin, the most abundant reported hosts are human, followed by cattle, and plants. Among the 19 potentially pathogenic bacteria present only in the gut samples are

*Brachyspira pilosicoli*, *Campylobacter coli* and *Campylobacter jejuni* strains, some strains of *C. difficile* and *E. coli*, *Salmonella enterica* strains, and some *Shigella* (*S. boydii, S. dysenteriae,* and *S. flexneri*). And among those 50 present only in the facial skin dataset, we found various strains of *Acinetobacter baumannii*, *Actinobacillus pleuropneumoniae*, *Burkholderia* spp., *Capnocytophaga gingivalis* ATCC 33624, some *Vibrio* (*V. harveyi* HY01, *V. ordalii* ATCC 33509, *V. shilonii* AK1, *V. splendidus* 12B01, and *V. tasmaniensis* ZS-17), and various *Xanthomonas* spp., among others.

***Fusobacteria and Clostridia pathogenicity***

It has been speculated that the large abundance of Fusobacteria and Clostridia in the vulture gut outcompetes other more virulent and toxic relatives, being harmless pathogenic versions that occupy the space and resources that more pathogenic versions would occupy otherwise [20]. To examine this hypothesis, we searched for toxin-related genes from these taxa in the gut functional core. We identified two putative enterotoxins from *C. perfringes*, and interestingly, also a protein in two gut samples from the bacteriocinogenic plasmid pIP404 from *C. perfringes* [21]; less toxin-related genes were found for *Fusobacterium*.

**Microbiome mediated protection**

***Probiotics and beneficial functions***

One of the most abundant taxa in the facial skin microbiome was *Pseudomonas fluorescens*, which can produce the antibiotic mupirocin [22]. This antibiotic is used for treating skin, ear, and eye disorders by interfering with isoleucyl-tRNA synthetase activity of pathogens, suggesting it may play a role in treating illnesses that some bacteria could cause on the vulture. Among the potentially health-beneficial bacteria identified in higher abundance in the facial skin microbiome was

*Arthrobacter phenanthrenivorans* (max. coverage in the facial samples= 4.4%, max. coverage in the gut samples= 0.4%, with 163 annotated genes in the facial skin and 66 annotated genes in the gut), which is able to degrade phenanthrene [23], a skin-irritating poly-cyclic aromatic hydrocarbon. Also, part of the facial functional core that takes taxonomy into account is *Hylemonella gracilis* (max. coverage= ~4%, 69 assembled genes), which has been shown to prevent long term colonization by *Yersinia pestis* [24]. Among the plasmids present only or most abundantly in the gut microbiome, we identified those of probiotic bacteria *Lactobacillus brevis* KB290 [25], *L. casei* W56 [26], *L. paracasei* [27], *L. salivarius* CECT 5713 [28], an *L. reuteri* SD2112 [29], which produces the antimicrobial reuterin [30]. We also identified plasmids from *Lactobacillus sakei* (max. coverage in the gut samples= 91.5%, max. coverage in the facial skin samples= 26.85%), from which we also identified a putative bacteriocin immunity protein and a type II secretion system protein coding gene in the NR gene catalogue. Folate has been related to skin cancer prevention [31]. Notably, one of the most abundant sub-pathways in the facial skin intra-sample comparison was the folate biosynthesis. In this regard, we also identified the gene dihydropteroate synthase type-2 from *Acinetobacter* sp. NIPH 899 in the facial microbiome. Furthermore, aminobenzoate is used to treat skin disorders, and we found that the aminobenzoate degradation subclass from the xenobiotics degradation metabolism was the most abundant in both facial skin and gut microbiomes.

***Antibiotics***

We identified several genes for the biosynthesis of antibiotics. Among the most abundant metabolic subclasses in both facial skin and gut microbiomes was the biosynthesis of carbapenem. From the metabolism of terpenoids and polyketides, the most abundant subclasses in the facial skin and gut microbiome were the biosynthesis of tetracycline, macrolides, and ansamycins. Also,

among those subclasses not driving functional variation in the facial skin microbiome were the biosynthesis of monobactam, anthocyanin, and ansamycins. Furthermore, in the facial skin MOCAT strict core, we identified a gene involved in the production of naphthocyclinones antibiotics [32] from *Herbaspirillum frisingense* [33].

***Phages***

In accordance to a potential phage therapy strategy, among the phages present only in the facial skin microbiome we identified the phage *phi* MR11, which eliminates multidrug resistant *Staphylococcus aureus* [34]. We also identified *Acinetobacter* phage Petty, which infects *Acinetobacter baumanii* [35], a multidrug resistant pathogen usually isolated from wounds. The phage Acibel004, active against *A. baumanii* [36], was also present only in the facial skin microbiome. From the facial skin functional strict core, the most abundant phage was BPP-1, which infects pathogenic *Bordetella* spp. [37]. Among the identifications was the *Enterobacteria* phage P22 present only in the gut samples, which infects the pathogenic *S. typhimurium* [38]. Furthermore, we identified the phage L-413C, which is specific for *Y. pestis* [39] and identified as more abundant in the gut samples. Also present was the phage *phi* CD119, which reduces toxin production in *C. difficile* [40]; notably, we also identified genes related to *C. difficile* virulence. Also, the *Enterobacteria* phage HK620 was identified as more abundant in the gut microbiome, this phage absorbs the O-antigen of *E. coli* H [41]. The gut functional strict core contains the phage phiCD6356, which infects *C. difficile* [42], and phage SPN3US, which has shown effective inhibition of *S. enterica* [43]. Notably, we identified putative virulent genes from *S. enterica* in the gut microbiome (Additional file 7).

***Defence versus eukaryotes***

Besides bacterial killing strategies, we also identified insecticide, fungicide, and antiparasitic related taxa and genes. Among those taxa significantly more abundant in the facial skin microbiome we identified *Lysinibacillus sphaericus*, which produces insecticidal toxins that control mosquito growth [44], and for which we assembled the gene coding for sphaericolysin, an insecticidal pore-forming toxin. We also identified *Pseudomonas entomophila* (8 genes in the gut samples, max. coverage in the gut samples= 0.2%, max. mapping reads in the gut samples= 216; 175 genes in the facial skin samples, max. coverage in the facial skin samples= 0.5%, max. mapping reads in the facial skin samples= 542) which infects insects causing lethality in fly larvae and adults [45,46]. Notably, we identified in the NR gene set catalogue a gene annotated as derived from *P. entomophila* coding for an insecticidal toxin SepC/Tcc class. We also identified *Streptomyces violaceusniger* (present in 20 facial skin samples, 0.1% max. coverage, 214 max mapping reads, 28 assembled genes; 11 gut samples, 0.03% max. coverage, 58 max mapping reads, 15 assembled genes), which is an antifungal for various plant fungal pathogens [47,48]. Among the antiparasitic taxa in the facial skin relaxed core, we identified *Kitasatospora setae*, which is capable of producing the antitrichomonal setamycin [49], and *Streptomyces bingchenggensis*, which produces the anthelmintic macrolide milbemycin [50]. We also identified *Heterorhabditis bacteriophora* (found in 34 gut samples and 24 facial skin samples), which kills pests like fleas, ants, and flies by releasing *Photorhabdus luminescens* bacteria from their digestive tract [51]. Interestingly, we also identified *P. luminescens* (max. mapping reads in the gut samples= 462, max. mapping reads in the facial skin= 20). Although present in low amounts, we also identified in 21 of the gut samples the *Dictyostelium* genus (*D. intermedium and D. citrium*, max. mapping reads in the gut samples= 596, max. mapping reads in the facial skin samples= 36), which is a bacteriovorous protozoa present in the soil, where they keep bacterial populations in balance [52].

We also identified *Adineta vaga*, which feeds on dead organic matter, mainly dead bacteria and protozoans [53], in 93.6% of the intestinal samples and 54.5% of the facial skin samples (gut normalized abundance= 54,230 mapping reads, facial skin normalized abundance= 7,972 mapping reads).

***Non-antibiotic mechanisms***

Among the taxa more abundant in the facial skin microbiome, it is interesting to note the identification of bacteria capable of growing in cancerogenous substances and producers of anti-cancer immunosuppressant substances. This is of relevance given that such bacteria might produce antimicrobial alternatives or products beneficial to the vulture to aid in fighting the constant aggression of the toxins present in the carcasses. Present in the relaxed facial skin core we identified *Chromobacterium violaceum* [54] and *Janthinobacterium* sp. HH01 [55], which produce violacein, an anticancer, antibacterial, antifungal, and antiviral compound. *Janthinobacterium* sp. HH01 was also present in the facial skin functional strict core. The bacteria *Polaromonas naphthalenivorans*, capable of degrading the potentially carcinogenic naphthalene [56], was present more abundantly in the facial skin microbiome (max. mapping reads in facial skin samples= 3,194, max. mapping reads gut samples= 60). This is of relevance given that 1-methyl naphthalene is produced in carrion decomposition [57,58].

Biosurfactants represent a strong antimicrobial means of blind killing, including bacteria with antibiotic resistance that would otherwise be difficult to treat. Interestingly, we identified the biosurfactant producer fungi *Yarrowia lipolytica* [59] and the bacteria *Rhodococcus erythropolis* [60] in both facial skin and gut microbiomes. We also identified surfactin biosynthesis regulatory proteins from *Flavobacteriaceae*. Furthermore, annotation of the non-mapping reads with DIAMOND identified various surfactin synthetase proteins from various genera in the facial skin

microbiome. Surfactin is a very powerful surfactant that serves as antibacterial, antiviral, antifungal, and attacks red blood cells with deadly efficiency [61,62].

***Biofilm and colonization resistance***

The presence of biofilm forming bacteria has been suggested to play a protective role for the host [63]. We identified the biofilm-forming bacteria *P. fluorescens* [64] in higher abundance in the facial skin and as part of the strict taxonomic facial skin microbiome core. Interestingly, in the gut functional core, we identified genes coding for biofilm formation promoter proteins from *F. mortiferum*, such as sialic acid-binding periplasmic protein [65], and rubrerythrin [66]. And from *C. perfringens*, such as UDP-glucuronic acidepimerase [67], putative alginate biosynthesis protein AlgI [68], and fibronectin-binding protein [69], as well as toxin-antitoxin biofilm protein from *E. coli* [70]. These results suggest that potentially pathogenic bacteria could form biofilms which allow them to thrive in the gut.

***Pathogenic biofilm formation***

Notably, a biofilm-mediated protection scenario requires a special interaction with the vulture's immune response, otherwise a scenario such as that in the biofilm formation in patients with cystic fibrosis (CF) would develop. The biofilm in CF patients results in clinical symptoms due to the host immune response producing tissue damage as a result of the chronic inflammation mediated by the immune complex that is trying to attack the highly resistant bacteria in the biofilm [71]. Thus, the colonization resistance mechanism of the vulture microbiome mediated by the biofilm formation requires that the vulture's immune system does not react against with a chronic inflammatory response. Interestingly, the *PIK3AP1* and *TNFAIP3* genes, involved in B-cell development, antigen presentation, auto-inflammation, and NF-kappa B activation, have been found to contain potentially functional altering amino acid changes in the cinereous vulture

(*Aegypius monachus*) [72]. Even more, in CF patients it has been shown that sub-minimal inhibitory concentrations of some antibiotics, such as erythromycin (from which we identified related genes in our NR gene set) and azithromycin, suppress the production of exoproducts, such as proteases and phospholipase C [73–75]. The inhibition of these exoproducts reduces the antigenic load and thus could lead to the decrease of immune system response. Similar modulatory mechanisms could be taking place in the vulture gut microbiome, where we identified various antibiotics.

**Resistance genes**

In the ResFinder database search, we identified resistance genes in 17 samples out of the 33 facial skin samples (min= 6 genes, 1$^{st}$ Qu= 18 genes, median= 36 genes, $\bar{x}$= 44.35 genes, 3$^{rd}$ Qu= 72 genes, max= 107 genes), and in 36 samples out of the 46 gut samples (min= 6 genes, 1$^{st}$ Qu= 10 genes, median= 15 genes, $\bar{x}$= 19.3 genes, 3$^{rd}$ Qu= 25 genes, max= 59 genes), totalling 215 genes (166 in the facial skin, and 139 in the gut) against 15 substances (15 in the facial skin, and 14 in the gut). There is no statistical difference in the number of substances with resistance genes identified by vulture species ($P$= 0.30, $\bar{x}_{C.\ atratus}$= 4.175, $\bar{x}_{C.\ aura}$= 3.405), neither by sample type ($P$= 0.92, $\bar{x}_{gut}$= 3.84, $\bar{x}_{facial\ skin}$= 3.76), however there is a difference in the abundance, being more abundant in the gut microbiome ($P$= 0.018, $\bar{x}_{gut}$ = 88,016.8, $\bar{x}_{facial\ skin}$= 56,985.7). The one substance with resistance genes in the most number of samples (52) is tetracycline, and there is no substance with resistance genes in more than 90% of the samples. In at least 50% of the facial skin and gut samples there were resistance genes for aminoglycoside, lincosamide, macrolides, and tetracycline. Only facial skin samples were found to contain resistance genes against

sulphonamide. Interestingly, in the gut dataset there was an abundance of genes resistant to lincosamide, which is used to treat pseudomembranous colitis caused by *C. difficile* [76].

Searching against the Resfams database, we identified 170 different resistance genes in the facial skin, and 170 in the gut, totalling 170 unique resistance proteins. Samples have a minimum of 0 genes coding for resistance proteins (one facial skin sample) and a maximum of 170 (4 gut samples and 2 facial skin samples), with $\bar{x}$= 104.3 per sample, with each gene coding for a resistance protein being present in a mean number of 47.23 samples. There was a significant difference in the number of identified genes coding for proteins with resistance to antibiotics between facial skin and gut (*P*= 0.049, $\bar{x}_{facial\ skin}$= 87.3, $\bar{x}_{gut}$= 117.0), although there was no statistical difference in the abundance (*P*= 0.69). The protein present in the most number of samples (69 samples) was from the phosphotransferase enzyme family, which confers resistance to various aminoglycosides [77]. The resistance genes identified from the ResFams search can be classified as resistant to the following types of drugs: *i*) for treatment of various diseases, such as urinary and respiratory diseases, meningitis, tuberculosis, and against *Staphylococcus* and *Streptococcus*, *ii*) for the treatment of enteric diseases, and *iii*) for the treatment of other diseases caused by fungi or protozoa. Interestingly, there were also genes resistant to indiscriminate antibiotics, such as surfactants, organic solvents, heavy metal ions, antifolates, and carcinogens and anticarcinogens.

Among the most abundant genes from the facial skin MOCAT NR gene set we found antibiotic resistance genes for aminoglycoside from *A. baumanii*, and kanamycin from *Staphylococcus epidermidis*. In the search of the facial skin dataset against the ResFinder database we identified a differentially abundant number of resistance genes to macrolide. Given that some macrolides have antibiotic or antifungal activity [78,79], their higher abundance in the facial skin is expected taking into account that the facial skin has a significantly greater fungal diversity (*P*= 0.029), with many

of them being plant pathogens. The facial skin microbiome also contained more resistance genes towards phenicol than the gut microbiome ($\overline{x}_{facial\ skin}$= 112,149.94, $\overline{x}_{gut}$= 2,177.54). Their use against infections in body parts such as eye and ear [80] could explain their higher abundance in the facial skin microbiome. In the ResFams database search we also identified resistance genes to drugs for the treatments of diseases such as enteric diseases (e.g. streptogramin [81] and bicyclomycin [82]), and tuberculosis (e.g. aminoglycoside [83] and oxazolidinones [84]). Interestingly, many of the antibiotics with a resistance gene would also pose serious adversities to the vulture, such as macrolides [85] and cephalosporin [86], which cause digestive disturbances to humans.

**References**

1. Lattanzio V, Lattanzio VMT, Cardinali A. Role of polyphenols in the resistance mechanisms of plants against fungal pathogens and insects. In: Phytochemistry: Advances in research. Research Signpost. 2006;23-67.
2. Falcone Ferreyra ML, Rius SP, Casati P. Flavonoids: biosynthesis, biological functions, and biotechnological applications. Front Plant Sci. 2012;3:222.
3. Vogt T. Phenylpropanoid biosynthesis. Mol Plant. 2010;3:2-20.
4. Dent BB, Forbes SL, Stuart BH. Review of human decomposition processes in soil. Environ Geol. 2004;45:576-85.
5. Evans WED. The chemistry of death. Charles C. Thomas Publisher. 1960.
6. Mayr D, Margesin R, Klingsbichel E, Hartungen E, Jenewein D, Schinner F, et al. Rapid detection of meat spoilage by measuring volatile organic compounds by using proton transfer reaction mass spectrometry. Appl Environ Microbiol. 2003;69:4697-705.

7. Phan N-T, Kim K-H, Jeon E-C, Kim U-H, Sohn JR, Pandey SK. Analysis of volatile organic compounds released during food decaying processes. Environ Monit Assess. 2012;184:1683-92.

8. Pati A, Abt B, Teshima H, Nolan M, Lapidus A, Lucas S, et al. Complete genome sequence of *Cellulophaga lytica* type strain (LIM-21). Stand Genomic Sci. 2011;4:221-32.

9. Bertolini JM, Rohovec JS. Electrophoretic detection of proteases from different *Flexibacter columnaris* strains and assessment of their variability. Dis Aquat Organ. 1992;12:121-8.

10. Declercq AM, Haesebrouck F, Van den Broeck W, Bossier P, Decostere A. Columnaris disease in fish: a review with emphasis on bacterium-host interactions. Vet Res. 2013;44:27.

11. Ramezani M, MacIntosh SE, White RL. Utilization of D-amino acids by *Fusobacterium nucleatum* and *Fusobacterium varium*. Amino Acids. 1999;17:185-93.

12. Wahren A, Holme T. Amino acid and peptide requirement of *Fusiformis necrophorus*. J Bacteriol. 1973;116:279-84.

13. Budd A, Blandin S, Levashina EA, Gibson TJ. Bacterial alpha2-macroglobulins: colonization factors acquired by horizontal gene transfer from the metazoan genome? Genome Biol. 2004;5:R38.

14. Cáceres LC, Bonacci GR, Sánchez MC, Chiabrando GA. Activated α(2) macroglobulin induces matrix metalloproteinase 9 expression by low-density lipoprotein receptor-related protein 1 through MAPK-ERK1/2 and NF-κB activation in macrophage-derived cell lines. J Cell Biochem. 2010;111:607-17.

15. Lin AH-M, Nichols BL, Quezada-Calvillo R, Avery SE, Sim L, Rose DR, et al.

Unexpected high digestion rate of cooked starch by the Ct-maltase-glucoamylase small intestine mucosal α-glucosidase subunit. PLoS One. 2012;7:e35473.

16. Slaughter SL, Ellis PR, Butterworth PJ. An investigation of the action of porcine pancreatic alpha-amylase on native and gelatinised starches. Biochim Biophys Acta. 2001;1525:29-36.

17. Wong SG, Dessen A. Structure of a bacterial α2-macroglobulin reveals mimicry of eukaryotic innate immunity. Nat Commun. 2014;5:4917.

18. Stevens DL, Bryant AE. The role of clostridial toxins in the pathogenesis of gas gangrene. Clin Infect Dis. 2002;35:S93-100.

19. Brooke JS. *Stenotrophomonas maltophilia*: an emerging global opportunistic pathogen. Clin Microbiol Rev. 2012;25:2-41.

20. Roggenbuck M, Bærholm Schnell I, Blom N, Bælum J, Bertelsen MF, Pontén TS, et al. The microbiome of New World vultures. Nat Commun. 2014;5:5498.

21. Garnier T, Cole ST. Complete nucleotide sequence and genetic organization of the bacteriocinogenic plasmid, pIP404, from *Clostridium perfringens*. Plasmid. 1988;19:134-50.

22. El-Sayed AK, Hothersall J, Cooper SM, Stephens E, Simpson TJ, Thomas CM. Characterization of the mupirocin biosynthesis gene cluster from *Pseudomonas fluorescens* NCIMB 10586. Chem Biol. 2003;10:419-30.

23. Kallimanis A, Frillingos S, Drainas C, Koukkou AI. Taxonomic identification, phenanthrene uptake activity, and membrane lipid alterations of the PAH degrading *Arthrobacter* sp. strain Sphe3. Appl Microbiol Biotechnol. 2007;76:709-17.

24. Pawlowski DR, Raslawsky A, Siebert G, J Metzger D. Identification of *Hylemonella*

*gracilis* as an antagonist of *Yersinia pestis* persistence. J Bioterror Biodef. 2011;2.

25. Murakami K, Habukawa C, Nobuta Y, Moriguchi N, Takemura T. The effect of *Lactobacillus brevis* KB290 against irritable bowel syndrome: a placebo-controlled double-blind crossover trial. Biopsychosoc Med. 2012;6:16.

26. Hochwind K, Weinmaier T, Schmid M, van Hemert S, Hartmann A, Rattei T, et al. Draft genome sequence of *Lactobacillus casei* W56. J Bacteriol. 2012;194:6638.

27. Bendali F, Madi N, Sadoun D. Beneficial effects of a strain of *Lactobacillus paracasei* subsp. paracasei in *Staphylococcus aureus*-induced intestinal and colonic injury. Int J Infect Dis. 2011;15:e787-94.

28. Martín R, Jiménez E, Olivares M, Marín ML, Fernández L, Xaus J, et al. *Lactobacillus salivarius* CECT 5713, a potential probiotic strain isolated from infant feces and breast milk of a mother-child pair. Int J Food Microbiol. 2006;112:35-43.

29. Cadieux P, Wind A, Sommer P, Schaefer L, Crowley K, Britton RA, et al. Evaluation of reuterin production in urogenital probiotic *Lactobacillus reuteri* RC-14. Appl Environ Microbiol. 2008;74:4645-9.

30. Casas IA, Dobrogosz WJ. Validation of the probiotic concept: *Lactobacillus reuteri* confers broad-spectrum protection against disease in humans and animals. Microb Ecol Heal Dis. 2000;12:247-285.

31. Williams JD, Jacobson EL, Kim H, Kim M, Jacobson MK. Folate in skin cancer prevention. Subcell Biochem. 2012;56:181-97.

32. Brünke P, Sterner O, Bailey JE, Minas W. Heterologous expression of the naphthocyclinone hydroxylase gene from *Streptomyces arenae* for production of novel hybrid polyketides. Antonie Van Leeuwenhoek. 2001;79:235-45.

33. Straub D, Rothballer M, Hartmann A, Ludewig U. The genome of the endophytic bacterium *H. frisingense* GSF30(T) identifies diverse strategies in the *Herbaspirillum* genus to interact with plants. Front Microbiol. 2013;4:168.

34. Rashel M, Uchiyama J, Ujihara T, Uehara Y, Kuramoto S, Sugihara S, et al. Efficient elimination of multidrug-resistant *Staphylococcus aureus* by cloned lysin derived from bacteriophage phi MR11. J Infect Dis. 2007;196:1237-47.

35. Mumm IP, Wood TL, Chamakura KR, Kuty Everett GF. Complete genome of *Acinetobacter baumannii* podophage Petty. Genome Announc. 2013; doi:10.1128/genomeA.00850-13.

36. Merabishvili M, Vandenheuvel D, Kropinski AM, Mast J, De Vos D, Verbeken G, et al. Characterization of newly isolated lytic bacteriophages active against *Acinetobacter baumannii*. PLoS One. 2014;9:e104853.

37. Liu M, Deora R, Doulatov SR, Gingery M, Eiserling FA, Preston A, et al. Reverse transcriptase-mediated tropism switching in *Bordetella* bacteriophage. Science. 2002;295:2091-4.

38. Kwoh DY, Kemper J. Bacteriophage P22-mediated specialized transduction in *Salmonella typhimurium*: high frequency of aberrant prophage excision. J Virol. 1978;27:519-34.

39. Garcia E, Chain P, Elliott JM, Bobrov AG, Motin VL, Kirillina O, et al. Molecular characterization of L-413C, a P2-related plague diagnostic bacteriophage. Virology. 2008;372:85-96.

40. Govind R, Vediyappan G, Rolfe RD, Dupuy B, Fralick JA. Bacteriophage-mediated toxin gene regulation in *Clostridium difficile*. J Virol. 2009;83:12037-45.

41. Barbirz S, Müller JJ, Uetrecht C, Clark AJ, Heinemann U, Seckler R. Crystal structure of

*Escherichia coli* phage HK620 tailspike: podoviral tailspike endoglycosidase modules are evolutionarily related. Mol Microbiol. 2008;69:303-16.

42. Horgan M, O'Sullivan O, Coffey A, Fitzgerald GF, van Sinderen D, McAuliffe O, et al. Genome analysis of the *Clostridium difficile* phage PhiCD6356, a temperate phage of the *Siphoviridae* family. Gene. 2010;462:34-43.

43. Kagawa H, Ono N, Enomoto M, Komeda Y. Bacteriophage chi sensitivity and motility of *Escherichia coli* K-12 and *Salmonella typhimurium* Fla- mutants possessing the hook structure. J Bacteriol. 1984;157:649-54.

44. Berry C. The bacterium, *Lysinibacillus sphaericus*, as an insect pathogen. J Invertebr Pathol. 2012;109:1-10.

45. Vodovar N, Vallenet D, Cruveiller S, Rouy Z, Barbe V, Acosta C, et al. Complete genome sequence of the entomopathogenic and metabolically versatile soil bacterium *Pseudomonas entomophila*. Nat Biotechnol. 2006;24:673-9.

46. Vodovar N, Vinals M, Liehl P, Basset A, Degrouard J, Spellman P, et al. *Drosophila* host defense after oral infection by an entomopathogenic *Pseudomonas* species. Proc Natl Acad Sci. 2005;102:11414-9.

47. Höltzel A, Kempter C, Metzger JW, Jung G, Groth I, Fritz T, et al. Spirofungin, a new antifungal antibiotic from *Streptomyces violaceusniger* Tü 4113. J Antibiot. 1998;51:699-707.

48. Kang MJ, Strap JL, Crawford DL. Isolation and characterization of potent antifungal strains of the *Streptomyces violaceusniger* clade active against *Candida albicans*. J Ind Microbiol Biotechnol. 2010;37:35-41.

49. Ichikawa N, Oguchi A, Ikeda H, Ishikawa J, Kitani S, Watanabe Y, et al. Genome

sequence of *Kitasatospora setae* NBRC 14216T: an evolutionary snapshot of the family *Streptomycetaceae*. DNA Res. 2010;17:393-406.

50. Wang X-J, Yan Y-J, Zhang B, An J, Wang J-J, Tian J, et al. Genome sequence of the milbemycin-producing bacterium *Streptomyces bingchenggensis*. J Bacteriol. 2010;192:4526-7.

51. Chattopadhyay A, Bhatnagar NB, Bhatnagar R. Bacterial insecticidal toxins. Crit Rev Microbiol. 2004;30:33-54.

52. Landolt C. Dictyostelid cellular slime molds from caves. J Cave Karst Stud. 2006;68:22-6.

53. Flot J-F, Hespeels B, Li X, Noel B, Arkhipova I, Danchin EGJ, et al. Genomic evidence for ameiotic evolution in the bdelloid rotifer *Adineta vaga*. Nature. 2013;500:453-7.

54. Hoshino T. Violacein and related tryptophan metabolites produced by *Chromobacterium violaceum*: biosynthetic mechanism and pathway for construction of violacein core. Appl Microbiol Biotechnol. 2011;91:1463-75.

55. Hornung C, Poehlein A, Haack FS, Schmidt M, Dierking K, Pohlen A, et al. The *Janthinobacterium* sp. HH01 genome encodes a homologue of the *V. cholerae* CqsA and *L. pneumophila* LqsA autoinducer synthases. PLoS One. 2013;8:e55045.

56. Jeon CO, Park W, Ghiorse WC, Madsen EL. *Polaromonas naphthalenivorans* sp. nov., a naphthalene-degrading bacterium from naphthalene-contaminated sediment. Int J Syst Evol Microbiol. 2004;54:93-7.

57. Vass AA, Smith RR, Thompson C V, Burnett MN, Wolf DA, Synstelien JA, et al. Decompositional odor analysis database. J Forensic Sci. 2004;49:760-9.

58. Forbes SL, Perrault KA. Decomposition odour profiling in the air and soil surrounding vertebrate carrion. PLoS One. 2014;9:e95107.

59. Fontes GC, Ramos NM, Amaral PFF, Nele M, Coelho MAZ. Renewable resources for biosurfactant production by *Yarrowia lipolytica*. Brazilian J Chem Eng. 2012;29:483-94.

60. Pacheco GJ, Ciapina EMP, Gomes E de B, Junior NP. Biosurfactant production by *Rhodococcus erythropolis* and its application to oil removal. Braz J Microbiol. 2010;41:685-93.

61. Das P, Mukherjee S, Sen R. Genetic regulations of the biosynthesis of microbial surfactants: an overview. Biotechnol Genet Eng Rev. 2008;25:165-85.

62. Mor A. Peptide-based antibiotics: A potential answer to raging antimicrobial resistance. Drug Dev Res. 2000;50:440-7.

63. Mertz PM, Eaglstein WH. The effect of a semiocclusive dressing on the microbial population in superficial wounds. Arch Surg. 1984;119:287-9.

64. O'Toole GA, Kolter R. Initiation of biofilm formation in *Pseudomonas fluorescens* WCS365 proceeds via multiple, convergent signalling pathways: a genetic analysis. Mol Microbiol. 1998;28:449-61.

65. Swords WE, Moore ML, Godzicki L, Bukofzer G, Mitten MJ, VonCannon J. Sialylation of lipooligosaccharides promotes biofilm formation by nontypeable *Haemophilus influenzae*. Infect Immun. 2004;72:106-13.

66. Jiao Y, D'haeseleer P, Dill BD, Shah M, Verberkmoes NC, Hettich RL, et al. Identification of biofilm matrix-associated proteins from an acid mine drainage microbial community. Appl Environ Microbiol. 2011;77:5230-7.

67. Schmid J, Sieber V, Rehm B. Bacterial exopolysaccharides: biosynthesis pathways and engineering strategies. Front Microbiol. 2015;6:496.

68. Rehm BH, Valla S. Bacterial alginates: biosynthesis and applications. Appl Microbiol

Biotechnol. 1997;48:281-8.

69. McCourt J, O'Halloran DP, McCarthy H, O'Gara JP, Geoghegan JA. Fibronectin-binding proteins are required for biofilm formation by community-associated methicillin-resistant *Staphylococcus aureus* strain LAC. FEMS Microbiol Lett. 2014;353:157-64.

70. Kim Y, Wang X, Ma Q, Zhang X-S, Wood TK. Toxin-antitoxin systems in *Escherichia coli* influence biofilm formation through YjgK (TabA) and fimbriae. J Bacteriol. 2009;191:1258-67.

71. Høiby N, Krogh Johansen H, Moser C, Song Z, Ciofu O, Kharazmi A. *Pseudomonas aeruginosa* and the *in vitro* and *in vivo* biofilm mode of growth. Microbes Infect. 2001;3:23-35.

72. Chung O, Jin S, Cho YS, Lim J, Kim H, Jho S, et al. The first whole genome and transcriptome of the cinereous vulture reveals adaptation in the gastric and immune defense systems and possible convergent evolution between the Old and New World vultures. Genome Biol. 2015;16:215.

73. Sakata K, Yajima H, Tanaka K, Sakamoto Y, Yamamoto K, Yoshida A, et al. Erythromycin inhibits the production of elastase by *Pseudomonas aeruginosa* without affecting its proliferation *in vitro*. Am Rev Respir Dis. 1993;148:1061-5.

74. Molinari G, Guzmán CA, Pesce A, Schito GC. Inhibition of *Pseudomonas aeruginosa* virulence factors by subinhibitory concentrations of azithromycin and other macrolide antibiotics. J Antimicrob Chemother. 1993;31:681-8.

75. Kobayashi H. Airway biofilm disease: Clinical manifestations and therapeutic possibilities using macrolides. J Infect Chemother. 1995;1:1-15.

76. Mullany P, Wilks M, Tabaqchali S. Transfer of macrolide-lincosamide-streptogramin B

(MLS) resistance in *Clostridium difficile* is linked to a gene homologous with toxin A and is mediated by a conjugative transposon, Tn5398. J Antimicrob Chemother. 1995;35:305-15.

77. Sarwar M, Akhtar M. Cloning of aminoglycoside phosphotransferase (APH) gene from antibiotic-producing strain of *Bacillus circulans* into a high-expression vector, pKK223-3. Purification, properties and location of the enzyme. Biochem J. 1990;268:671-7.

78. Aszalos A, Bax A, Burlinson N, Roller P, McNeal C. Physico-chemical and microbiological comparison of nystatin, amphotericin A and amphotericin B, and structure of amphotericin A. J Antibiot. 1985;38:1699-713.

79. Zotchev SB. Polyene macrolide antibiotics and their applications in human therapy. Curr Med Chem. 2003;10:211-23.

80. Bron AJ, Leber G, Rizk SN, Baig H, Elkington AR, Kirkby GR, et al. Ofloxacin compared with chloramphenicol in the management of external ocular infection. Br J Ophthalmol. 1991;75:675-9.

81. Johnston NJ, Mukhtar TA, Wright GD. Streptogramin antibiotics: mode of action and resistance. Curr Drug Targets. 2002;3:335-44.

82. Kohn H, Widger W. The molecular basis for the mode of action of bicyclomycin. Curr Drug Targets Infect Disord. 2005;5:273-95.

83. Investigation MRC. Streptomycin treatment of pulmonary tuberculosis. Br Med J. 1948;2:769-82.

84. Cynamon MH, Klemens SP, Sharpe CA, Chase S. Activities of several novel oxazolidinones against *Mycobacterium tuberculosis* in a murine model. Antimicrob Agents Chemother. 1999;43:1189-91.

85. Periti P, Mazzei T, Mini E, Novelli A. Adverse effects of macrolide antibacterials. Drug Saf. 1993;9:346-64.

86. Norrby SR. Side effects of cephalosporins. Drugs. 1987;34 Suppl 2:105-20.
